# An Approach to Modeling and Developing Virtual Sensors Used in the Simulation of Autonomous Vehicles

**DOI:** 10.3390/s25113338

**Published:** 2025-05-26

**Authors:** István Barabás, Calin Iclodean, Horia Beles, Csaba Antonya, Andreia Molea, Florin Bogdan Scurt

**Affiliations:** 1 Department of Automotive Engineering and Transports, Technical University of Cluj-Napoca Romania, Muncii Bd. 103-105, 400114 Cluj-Napoca, Romania; istvan.barabas@auto.utcluj.ro (I.B.); andreia.molea@auto.utcluj.ro (A.M.); 2 Department of Mechanical Engineering and Automotive, University of Oradea, Universitatii Str. 1, Oradea, 7, 410087 Oradea, Romania; scurt.florinbogdan@didactic.uoradea.ro; 3 Department of Automotive and Transport Engineering, Transilvania University of Brasov, Eroilor Bd. 29, 500036 Brasov, Romania; antonya@unitbv.ro

**Keywords:** virtual sensors, ideal sensor, Hi-Fi sensor, RSI sensor, virtual vehicle model, autonomous driving, deep learning, machine learning

## Abstract

A virtual model enables the study of reality in a virtual environment using a theoretical model, which is a digital image of a real model. The complexity of the virtual model must correspond to the reality of the evaluated system, becoming as complex as necessary and nevertheless as simple as possible, allowing for computer simulation results to be validated by experimental measurements. The virtual model of the autonomous vehicle was created using the CarMaker software package version 12.0, which was developed by the IPG Automotive company and is extensively used in both the international academic community and the automotive industry. The virtual model simulates the real-time operation of a vehicle’s elementary systems at the system level and provides an open platform for the development of virtual test scenarios in the application areas of autonomous vehicles, ADAS, Powertrain, and vehicle dynamics. This model included the following virtual sensors: slip angle sensor, inertial sensor, object sensor, free space sensor, traffic sign sensor, line sensor, road sensor, object-by-line sensor, camera sensor, global navigation sensor, radar sensor, lidar sensor, and ultrasonic sensor. Virtual sensors can be classified based on how they generate responses: sensors that operate on parameters derived from measurement characteristics, sensors that operate on developed modeling methods, and sensors that operate on applications.

## 1. Introduction

Sensors are electronic devices that generate electrical signals in response to various environmental stimuli [1]. Sensors’ operating principles are determined by how information is recorded, and sensors can be classified as resistive, piezoelectric, capacitive, optical, magnetic, QTC (Quantum Tunneling Composite), triboelectric effect, FET (Field-Effect Transistor), and so on [2]. Solid-state sensors are integrated devices that preprocess an analog or digital signal before delivering a sensory response that embedded systems can process.

A virtual sensor is a software emulation of a physical sensor that uses real data, mathematical models, fuzzy logic, neural networks, genetic algorithms, and ML (Machine Learning) and AI (artificial intelligence) models to estimate parameter values and anticipate scenarios [3]. ML algorithms comprise two types of mechanisms: interactive learning, in which the virtual sensor identifies relevant elements in data streams, and automatic teaching, in which the human factor proactively contributes relevant elements to training the learning process [4]. Virtual sensors are data-driven prediction models that identify the physical characteristics of the system into which they are integrated, providing a viable substitute for real sensors while also being a cost-effective solution [5,6,7,8,9]. A key advantage of virtual sensors lies in their ability to significantly reduce overall vehicle mass—not only by replacing physical sensors, but also by eliminating the associated wiring, insulators, and connectors needed to interface with embedded systems. This weight reduction contributes to enhanced fuel efficiency or extended range in electric vehicles, ultimately resulting in lower greenhouse gas emissions. These benefits underscore both the environmental and economic advantages of virtual sensing technologies. Such considerations are evident in models developed by companies producing autonomous shuttle buses (e.g., Apollo Baidu, EasyMile, Navya), which utilize electric vehicle platforms equipped with low-power engines (20–40 kW) and batteries with limited energy capacity (20–40 kWh) [10].

The methodology presented in this review enables the development of virtual sensors by abstracting physical sensor models and integrating empirical behavioral representations, thereby allowing output variables to be directly derived from input variables relevant to the monitored process.

Virtual sensor models are algorithms or mathematical models that estimate physical quantities using input data obtained from real sensors, statistical analysis, or input data generated by AI [11]. Virtual sensors are developed using software applications and do not require any additional or specific hardware components to function. According to [12], a virtual sensor is a software-only sensor (no hardware components) that can generate signals autonomously by integrating data/signals obtained synchronously or asynchronously from physical sensors or even other virtual sensors. Virtual sensor models developed by [13] and implemented in the software architecture of real vehicles include, for example, the tire wear virtual sensor and the brake wear virtual sensor, which monitor wear, lifespan, and potential anomalies during the operation of some of the vehicle’s systems and components (headlight levelling, tire pressure, tire temperature, e-motor temperature, brake temperature, suspension displacement, and so on) [14].

Virtual sensors offer a wide range of applications in vehicle active safety systems, including ABS (Antilock Braking System), AWD (All-Wheel Drive), ACC (Adaptive Cruise Control), and SAS (Smart Airbag System). The integration of virtual sensor parameters into the previously specified systems contributes to the management of the vehicle’s optimal operating state in order to improve functional performance and reduce fuel/energy consumption, which is correlated with a reduction in pollutant emissions [15].

Implementing virtual sensors on real vehicles improves the accuracy of monitored data while also expanding coverage to locations where physical sensors are unavailable. When integrated into an embedded system, virtual sensors perform preprocessing, error correction, merging, and optimization of input data sets. Essentially, virtual sensors utilize an algorithm or mathematical apparatus to process input data and produce high-complexity output data sets that match specified requirements, as demonstrated by Hu et al. [16].

Unlike physical sensors, which must be added to precise positions inside a vehicle’s structural architecture in order to function properly, virtual sensors depend on data sets collected from the vehicle’s embedded systems. Based on this information, virtual sensors calculate specified parameters without the need for extra hardware. The development of a virtual sensor necessitates the implementation of a functional algorithm for the system under consideration, which is based on a statistical model that reliably anticipates the essential parameters being studied [17]. Because virtual sensors are made up of software components, firmware upgrades can be accomplished remotely via the OTA (Over-The-Air) approach, eliminating the need for physical interventions to remove and install these sensors.

Virtual sensors may improve data accuracy and resolution by merging information from numerous sources (other sensors, electronic control units, actuator feedback) using advanced data fusion and processing algorithms [18,19]. These sensors might be very simple or extremely sophisticated, depending on the activities and consequences they simulate: stimulus, electrical requirements, ambient environment, operational restrictions, and functional safety [20]. However, the performance of virtual sensors could decrease with time because of changes in nonlinear dynamics and the complexity of physical processes in the environment, as well as nonlinear interactions between input and output variables [6,21]. Virtual sensors increase the accessibility of data from physical sensors, facilitating collaboration at the sensor, equipment, and organizational levels (allowing service providers to offer solutions based on the same hardware), allowing for more efficient use of the same hardware resources in interconnected systems, such as IoT (Internet of Things). Virtual sensors take data acquired by physical sensors and incorporate them into complicated software applications, where they are merged with other sources of information (databases) and processed by specialized algorithms to produce meaningful results [1,22].

**Figure 1:**
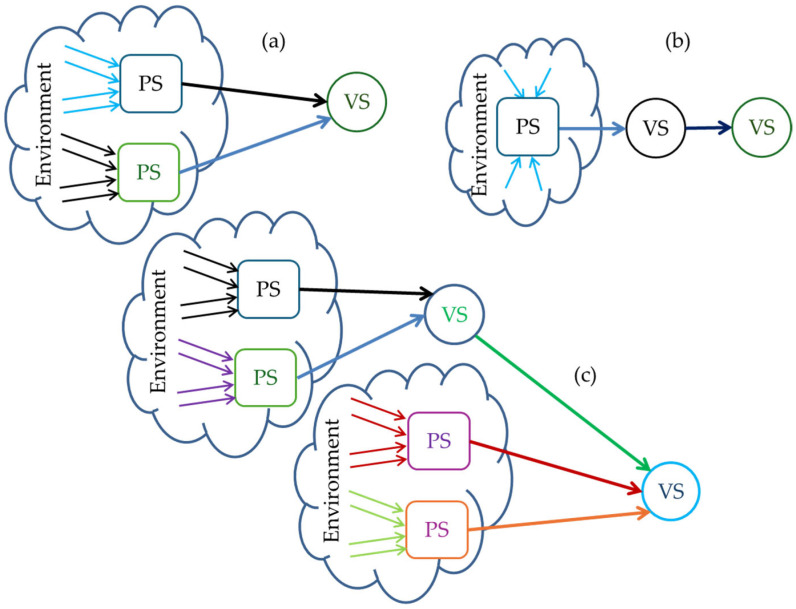
Different configurations of VS (virtual sensors) and PS (physical sensors). (**a**) Virtual sensors depend only on data from physical sensors (**b**) Virtual sensors depend entirely on information from other virtual sensors (**c**) Virtual sensors depend on data from both physical and virtual sensors.

Figure 1 illustrates three combinations of connected virtual and real sensors.

(a) Virtual sensors depend only on data from physical sensors. ESC (Electronic Stability Control) uses physical sensors like gyroscopes, accelerometers, wheel speed sensors, and virtual sensors to estimate the yaw/slip angle, allowing the vehicle to maintain control in low-grip conditions or dangerous turns.
(b) Virtual sensors depend entirely on information from other virtual sensors. In the case of FCW (Forward Collision Warning) and AEB (Automatic Emergency Braking), a virtual sensor is used to predict the trajectory of the vehicle and evaluate the distance to other vehicles.
(c) Virtual sensors depend on data from both physical and virtual sensors. This configuration can be found in the DMS (Driver Monitoring System), which uses physical sensors like a video camera and/or pressure sensors in the steering wheel and/or seat, and virtual sensors like those for estimating the driver’s level of attention and detecting the intention to leave the lane.

Finally, there is a requirement for using a suitable combination of physical and virtual sensors, in addition to maintaining functional algorithms for virtual sensors up to date [23].

Tactile Mobility [24] is a platform for monitoring, processing, and storing data from specific types of physical sensors that are installed in smart and interconnected vehicles in over 70 locations globally. This platform utilizes the data to create virtual sensors that, based on recorded scenarios, generate output parameters designed to improve the safety and performance of these vehicles [24]. The Tactile Mobility platform’s solution incorporates a software program into the vehicle’s built-in command and control systems, improving the operating regime by delivering information on road traffic, road conditions, tire grip and condition, vehicle mass, and so on.

Another platform that enables the use of virtual sensors in the automobile sector is the Compredict Virtual Sensor Platform [25], which calibrates, verifies, and implements these sensors on a wide range of real vehicle models. Thus, the Compredict platform can generate virtual models based on cloud-stored input data for the following virtual sensor categories: suspension travel, brake wear, brake temperature, wheel force transducer, vehicle mass, strain gauge, tire wear, tire pressure, tire temperature, LV (low-voltage) battery health, HV (high-voltage) battery health, and battery anomaly.

The development of virtual sensors is accelerating; according to a market study conducted by Mordor Intelligence for the period 2025–2030 in [26], the virtual sensor market will be worth 1.37 billion USD in 2025 and 5.35 billion USD by 2030. The advancement of smart manufacturing technologies, specifically the digitization of industrial processes in addition to the digitalization and validation of real vehicle models, contributes to the development of the virtual sensor sector.

Autonomous driving is one of the most innovative and rapidly evolving technologies in the automotive industry, with the potential to transform both personal and public transportation. It offers substantial benefits in road safety, traffic efficiency, and urban mobility. Central to this technological advancement are smart sensors, which are critical to enabling autonomous vehicles to operate safely and effectively. These sensors provide environmental perception, support real-time decision-making, and allow adaptive responses to dynamic traffic and weather conditions.

However, the deployment of smart sensors in autonomous systems presents several challenges, including high costs, hardware–software integration, cybersecurity risks, and a lack of component standardization.

This review investigates the transition from physical (hardware-based) sensors to virtual (software-based) sensors, drawing on a project conducted by the authors. This project involved digitizing real sensor models and implementing their functionalities within a virtual autonomous vehicle, thereby demonstrating the potential and practical application of virtual sensing in autonomous vehicle development. Virtual sensors are critical for modern vehicles in terms of improving autonomous driving capabilities, safety, and efficiency. Table 1 shows the sensors’ progression and their implementation in a vehicle’s constructive architecture from level 1 to level 5 automation (according to SAE J3016^TM^) [27,28]. The sensors used in the equipment of autonomous vehicles are constrained by their physical dimensions, mass, the necessity to be positioned in less accessible sections of the vehicle’s structural architecture, and the cost of these sensors [29]. It is evident that the number of sensors increases as automation levels rise, with ultrasonic sensors and Lidar 2D/3D indicating the most significant numerical increases.

## 2. Classification of the Virtual Sensor

### 2.1. Virtual Sensor Model

According to research teams, the key real sensors frequently utilized in the constructive architecture of autonomous vehicles, sensors that form the basis for defining the virtual sensors used in modeling the virtual vehicle model, are as follows [30]:

1. Camera sensors generate synthetic data on the recognition and classification of objects in the area [31,32,33], in addition to the vehicle’s positioning and orientation relative to close to objects and V2V (Vehicle-to-Vehicle) communication [34] based on the VLC (Visible Light Communication) principle [35]. The advantages of the camera sensor include the ability to provide data in real time, low latency in data acquisition and processing, adaptability to extreme lighting conditions (low lighting, bright lighting), accurate estimation of object position and orientation, and low production and implementation costs. The constraints of camera sensors include the need for a direct view of the surrounding objects, susceptibility to unexpected changes in lighting conditions, and the need for greater computer capacity due to the large quantities of data that are constantly generated.
2. Radar sensor generates data based on the reflection duration of radio waves ToF (Time of Flight) when detecting nearby target vehicles [36,37] and uses ML methods to estimate the current and future positions of nearby vehicles [38], respectively, using DL (deep learning) methods to avoid collisions [39]. The benefits of radar sensors include the capacity to provide the location of target vehicles in real time, flexibility to severe weather conditions (rain, snow, fog), and low manufacturing and installation costs. The constraints of radar sensors include the requirement for increased computer capacity due to the massive volumes of data generated on a continuous basis, as well as a reliance on extra hardware systems and software.
3. Lidar sensors provide a system based on generating a point cloud through 2D and 3D laser scanning for real-time localization of static and dynamic objects in proximity [40,41] and applies the YOLO (You Only Look Once) picture segmentation technique [42]. The advantages of lidar sensors include the ability to localize static and moving objects in proximity precisely. The disadvantages of lidar sensors include the need for greater computer power due to the large quantity of data generated continuously, sensitivity to bad weather conditions (rain, snow, fog), and high manufacturing and implementation costs.

Sensor fusion is the process of combining sensor signals [43,44] using CNN (convolutional neural network) neural networks, processing these signals with DL-type AI elements [45,46], detecting nearby objects in real time [47,48], and then making predictions about the evolution of these objects [49,50,51,52].

The virtual sensor models presented in this review were created, tested, and calibrated using the CarMaker simulation application from IPG Automotive, which is extensively used in the automotive industry for virtual vehicle model development at all stages. CarMaker is a platform that enables the development of any virtual test scenarios that are connected to other software applications [53].

Yeong et al. [54] classified physical and virtual sensors as smart or non-smart. Smart sensors are directly related to the IoT concept, and they are systems made up of interconnected devices that may collect and transport data remotely without the need for human involvement. A smart sensor is an IoT device that can condition and select incoming signals, process and interpret the generated data, and make decisions without the assistance of a separate processing unit [55].

Virtual sensors can be classed using the following criteria [56,57,58]:

1. Sensor fidelity could be classified as high-, medium-, or low-income.
2. Method for collecting information from the environment:
  (a) A deterministic strategy based on the simulation application’s mathematical apparatus and involving the usage of a vast volume of input parameters to represent the ideal behavior and response of the virtual sensor as accurately as possible;
  (b) A statistical technique based on statistical distribution functions, which include the normal, binomial, Poisson, or exponential distribution;
  (c) An electromagnetic field propagation approach to simulate electromagnetic wave propagation using Maxwell’s equations.
3. The objective of using sensors is to develop a vehicle’s operating mode based on observed metrics and to perform diagnostics using AI-based maintenance techniques to define the smart maintenance regime.

Virtual systems developed for simulation applications use additional virtual sensors that are intended to replace certain partial functionalities of the main real sensors to reduce the volume of input data, reduce computing power requirements, calibrate the main sensor, and provide an optimized output data stream [29].

**Figure 2:**
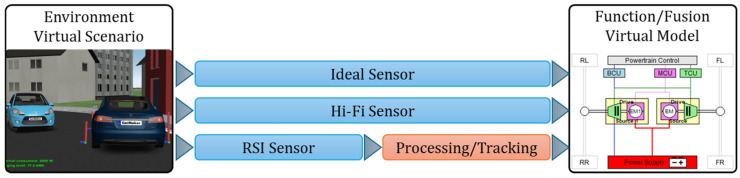
Virtual sensor classification.

CarMaker classifies virtual sensors into three types: ideal sensors, Hi-Fi (high-fidelity) sensors, and RSI (Raw Signal Interface) sensors (Figure 2). These virtual sensor models are intended to maximize the performance of the virtual vehicle model on which they are installed, as well as to assist the command-and-control system in developing and expanding the specific capabilities of each sensor to a higher class of sensors [59,60].

The virtual model developed in CarMaker incorporates the following command and control systems for advanced assistance functions: ADAS (Advanced Driver Assistance System): ACC, EBA (Emergency Brake Assist), LDW (Lane Departure Warning), LKA (Lane Keeping Assist), PA (Park Assist), ILA (Intelligent Light Assist), and TSA (Traffic Sign Assist). All these embedded systems evaluate and interpret data about the motor and/or vehicle’s operating mode by combining various virtual sensor models [61,62].

#### 2.1.1. Ideal Sensors

**Figure 3:**
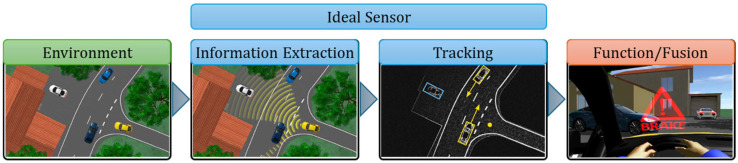
Ideal sensors.

The role of ideal sensors in the CarMaker simulation program is to collect information from the simulation environment and transmit it to an embedded system. Ideal sensors are virtual entities developed using software that are independent of technology (Figure 3) and equip the virtual vehicle model with the following: slip angle, inertial, object, free space, traffic sign, line, road, and object-by-line sensor. Physical impacts that occur in the real environment in the case of a model integrated into the HiL (Hardware-in-the-Loop) system have no effect on these ideal sensors that are integrated into the SiL (Software-in-the-Loop) model and do not generate information similar to a real sensor [63].

#### 2.1.2. Hi-Fi Sensors

**Figure 4:**
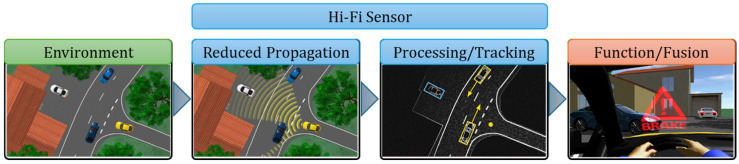
Hi-Fi sensors.

Hi-Fi sensors filter the information supplied to the embedded system and provide data on the physical impacts that occur in the real environment, particularly the detection and classification of static and dynamic objects in the area. The virtual vehicle model is equipped with the following Hi-Fi sensors (Figure 4): camera, global navigation, and radar sensors. Hi-Fi sensors have a role in reducing the impacts of false positives and false negatives that can occur in object perception and identification due to scenarios where part of the objects overlap, or environmental conditions prevent exact identification [64].

#### 2.1.3. RSI Sensors

**Figure 5:**
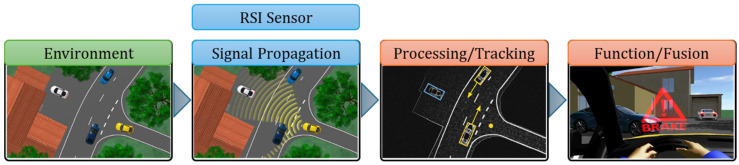
RSI sensors.

RSI sensors provide raw data and function identically to real sensors. The system filters, extracts, and interprets the data sent by the RSI sensors. Processing the information assigned by the RSI sensors necessitates high computational power, particularly for graphics processing provided by the GPU. There are RSI sensor types that conduct post-processing of the input in order to reduce the computing load on the embedded system. In the CarMaker simulation program, the RSI sensors identify objects in traffic and proximity, as well as all 3D surfaces in the surrounding environment. The utility IPGMovie, which is integrated with CarMaker, provides raw information for all these images. The virtual vehicle model is equipped with the following RSI sensors (Figure 5): ultrasonic RSI and lidar RSI [64].

The use of RSI sensors in a virtual environment requires modeling the properties of the materials that compose the objects in their vicinity, namely relative electric permittivity for electromagnetic waves and scattering effects. The direction and intensity of the field of waves reflected off 3D surfaces are significantly influenced by the material’s characteristic properties [65].

Figure 3, Figure 4 and Figure 5 illustrate the information cycle, which starts with extracting relevant details from the environment (green) and continues with processing and transmitting these data (blue) to embedded command and control systems (red).

**Figure 6:**
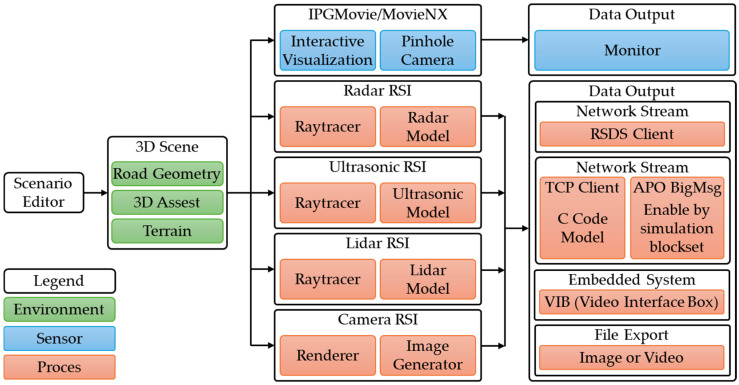
Interfaces and output format for RSI sensors.

RSI sensors process 3D images and offer real-time output for embedded systems, namely images and videos for the IPGMovie and/or MovieNX simulated scenario rendering system in CarMaker (Figure 6) [66].

### 2.2. Virtual Vehicle and Environmental Model

A virtual vehicle model is a prototype that precisely replicates the characteristics of the elements and systems of a real model using mathematical and physical models. After validating the model, the simulation method enables the virtual vehicle to run in any user-defined scenario in a short period of time and at a low cost [20,67].

A virtual vehicle model, also referred to as a DTw (Digital Twin), is a digital image of a physical vehicle. Renard et al. [68] define a DTw as an entity made up of a real model in a real space, a virtual model in a virtual space, and the data links that connect the real and virtual models. DTw systems, thanks to bidirectional communication, allow the virtual model to be updated when the real model’s state changes, and vice versa [69,70,71,72].

Tu et al. [73] argue that autonomous driving technology represents a technological revolution in transportation. It requires the integration of artificial intelligence and smart sensors, which—unlike human drivers—facilitate significantly faster and more intelligent decision-making.

**Figure 7:**
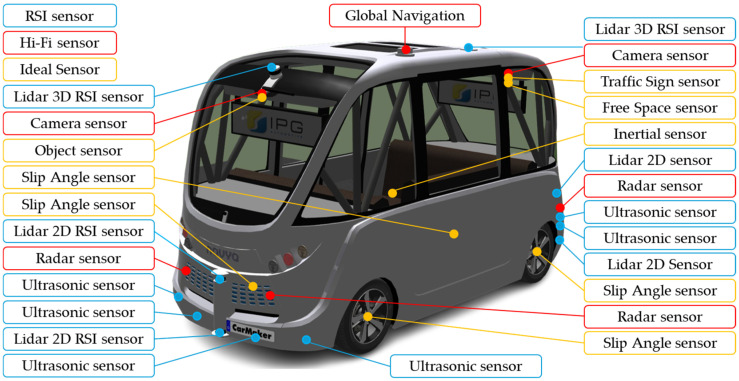
Virtual vehicle model for autonomous shuttle bus equipped with virtual sensors model: ideal sensors (yellow), Hi-Fi sensors (red), and RSI sensors (blue).

Navya Autonom^®^ Shuttle is an autonomous shuttle bus vehicle designed for public passenger transportation and based on the architecture of a fully electric vehicle. The Navya Autonom^®^ Shuttle was introduced by the French start-up Navya in October 2015, and the main technical requirements (navya.tech) were utilized for developing the virtual model in the CarMaker implementing virtual sensors (Figure 7) [10,27,74,75,76].

**Figure 8:**
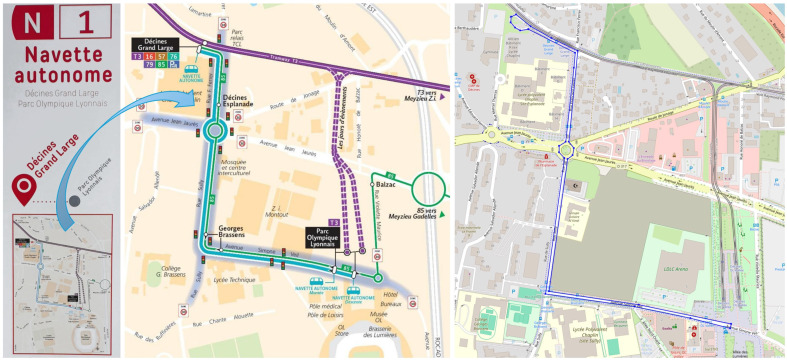
Virtual road vs. real road (TCL Lyon vs. GPSPrune—photo author (C.I.)).

The virtual environmental model consists of a virtual road and a virtual environment. The virtual road in which the virtual model of the autonomous vehicle travels was defined by digitizing the real route in Lyon, France, using geographical coordinates (latitude, longitude, and altitude) extracted from Google Earth (Figure 8) [27,77,78]. The digitized route was converted to SRTM (Shuttle Radar Topography Mission) coordinates [78] using the GPSPrune application [79]. The route with the altitude profile was loaded into CarMaker’s IPGRoad utility [80], which defined the following parameters in addition to the geographical coordinates (latitude, longitude, altitude): dimensions (length, width), connection angle, curvature, inclination, speed limit, and friction coefficient.

The virtual environment for the computer simulations was created using the CarMaker application’s Environment utility, which allowed the following atmospheric conditions to be defined: reference temperature, air density, air pressure, air humidity, cloud model, cloud intensity, fog, visibility, rain rate, wind velocity, and wind angle [62].

The autonomous driving system operating algorithm in CarMaker’s Vehicle Control section defines virtual driver behavior by addressing different driving styles corresponding to a human driver’s reaction speed and performance criteria under optimal energy consumption conditions [81].

## 3. Characteristics of the Virtual Sensor

### 3.1. Characteristics of Ideal Sensor

#### 3.1.1. Slip Angle Sensor

The Pacejka model is essential for realistic and thorough vehicle dynamics simulations, particularly when complex maneuvers and tire behavior are involved. It provides the necessary realism in illustrating tire forces, which are essential for vehicle motion. The Pacejka model is an useful mathematical model for simulating tire behavior in autonomous vehicle simulations in CarMaker because it accurately represents the complicated, nonlinear interaction between the tire and the road surface.

The slip angle sensor monitors the lateral slip angle between the steering wheel angle and the vehicle’s direction of motion. The slip angle sensor is located near the vehicle’s steering wheel [27,62,66].

The yaw angle is helpful in active safety systems because it controls cornering stability, prevents vehicle rollovers, and avoids lane departure. Controlling the yaw angle is required because a big yaw angle reduces the tires’ capacity to create lateral forces and greatly impairs the effectiveness of the vehicle control system. In addition to the yaw angle, the yaw rate is also a required variable for vehicle stability management [82,83].

**Figure 9:**
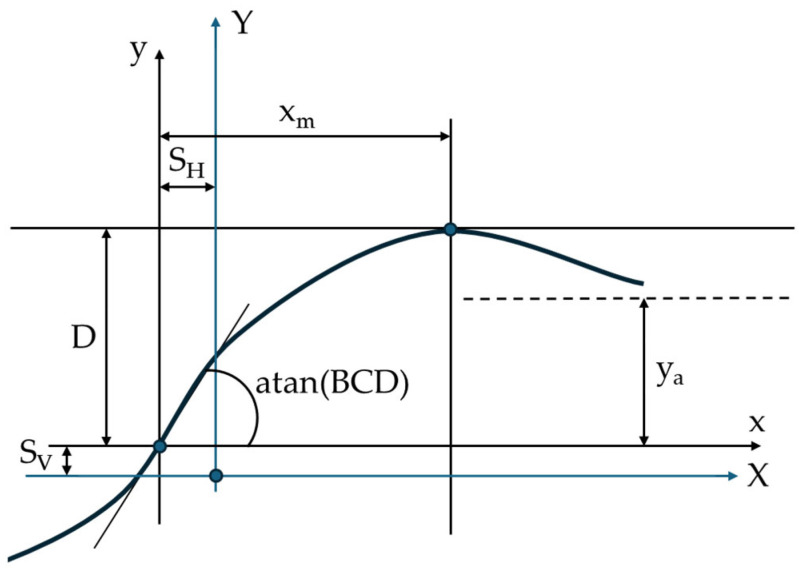
The Pacejka model.

The Pacejka model [84], a lateral force model, describes the complexity of the interaction between the tire and the road surface during dynamic maneuvers that are specific to autonomous vehicles. The Pacejka model is a semi-empirical mathematical model that represents the behavior of forces and moments created by a tire in contact with the road surface. It additionally provides a nonlinear representation of lateral forces, accounting for both large slip angles and normal forces. The Pacejka model (Figure 9) is commonly used in vehicle simulation and control, particularly for the development of advanced support systems and autonomous vehicles. The Pacejka model includes the following broad shape [83,84,85].

(1) $$y\left(x\right)=D\cdot sin\left\{C\cdot arctan\left[\left(1-E\right)Bx-E\cdot arctan\left(Bx\right)\right]\right\},$$

with

(2) $$Y\left(X\right)=y\left(x\right)+S_{V},$$

(3) $$x=X+S_{H},$$

where y(x) represents F_x_, F_y_, M_z_; B is the stiffness factor; C is the shape factor; D is the peak value; E is the curvature factor; S_H_ is the horizontal displacement; and S_V_ is the vertical displacement.

The Pacejka model generates a curve that passes through the origin (x = y = 0), reaches a maximum value, and then tends to a horizontal asymptote. To create a more accurate representation of tire behavior, the Pacejka model allows the curve’s position to be adjusted by inserting two translations, S_H_ and S_V_. These translations allow for the adjustment of potential asymmetries in the experimental data, resulting in a better fit between the model and reality. For the specific coefficients B, C, D, and E, the curve exhibits anti-symmetry with respect to the origin. The coefficient D specifies the peak value, whereas the product of B, C, and D determines the curve’s beginning slope. The coefficient C changes the operating range limits in the general formula, determining the shape of the curve. The stiffness factor is defined as the coefficient B, which is calculated using the inclination relative to the origin. The coefficient E is introduced to manage the curvature peak while also controlling its horizontal position. The shape factor C can be derived from the peak height and horizontal asymptote using the following formula [83,84,86]:

(4) $$C=1\pm \left(1-\frac{2}{\pi }arcsin\frac{y_{a}}{D}\right).$$

The curvature factor E is computed from B and C for the position x_m_ of the peak value using the following equation (if C > 1) [87]:

(5) $$E=\frac{Bx_{m}-tan\left[\frac{\pi }{\left(2C\right)}\right]}{Bx_{m}-\arctan\left(Bx_{m}\right)}.$$

The specific force is expressed as follows in both the longitudinal and transverse directions [88]:

(6) $$\Gamma =D\cdot sin\left\{C\cdot arctg\left[B\cdot s-E\cdot \left(B\cdot s-arctg\left(B\cdot s\right)\right)\right]\right\}.$$

The tire model parameters B, C, D, and E are all determined by the tire’s vertical load, which is defined as follows [89,90,91]:

(7) $$\begin{array}{c} F_{z1}=l_{r}\cdot \left(\frac{mg}{2l}+\frac{ma_{y}h}{2b_{f}l}\right)-\frac{ma_{x}h}{2l}F_{z2}=l_{r}\cdot \left(\frac{mg}{2l}-\frac{ma_{y}h}{2b_{f}l}\right)-\frac{ma_{x}h}{2l}F_{z3}\\ \;=l_{f}\cdot \left(\frac{mg}{2l}+\frac{ma_{y}h}{2b_{r}l}\right)+\frac{ma_{x}h}{2l}F_{z4}=l_{f}\cdot \left(\frac{mg}{2l}-\frac{ma_{y}h}{2b_{r}l}\right)+\frac{ma_{x}h}{2l} \end{array}$$

where F_z1_, F_z2_, F_z3_, and F_z4_ represent the vertical load of each corresponding tire, h is the height of the vehicle’s mass center, g is the acceleration of gravity, lf and lr are the distances from the vehicle’s gravity center to the front and rear axles, respectively, and bf and br are the half treads of the front and rear wheels. The sideslip angle of each tire can be defined as

(8) $$\begin{array}{c} a_{1}=\delta -arctan\frac{v_{y}+l_{f}\gamma }{v_{x}+b_{f}\gamma /2}a_{2}=\delta -arctan\frac{v_{y}+l_{f}\gamma }{v_{x}-b_{f}\gamma /2}a_{3}\\ \;=-arctan\frac{v_{y}-l_{r}\gamma }{v_{x}+b_{r}\gamma /2}a_{4}=-arctan\frac{v_{y}-l_{r}\gamma }{v_{x}-b_{r}\gamma /2} \end{array}$$

The tire slip rate can be obtained as follows:

(9) $$s_{j}=sgn(n_{j}r-v_{nj})\frac{\left(v_{nj},n_{i}r\right)-\left(v_{nj},n_{i}r\right)}{\left(v_{nj},n_{i}r\right)}$$

where s_j_ is the jth tire’s slip rate, n_j_ is its rotational speed, v_nj_ is its linear velocity, and r is its effective wheel radius.

To run the simulations in CarMaker, the virtual vehicle model dynamics library was utilized, which calculates the wheel slip angle using the force and moment equilibrium equations as the derivative of the virtual vehicle’s slip and yaw ratio [92,93]:

(10) $$\dot{Β}=-\gamma +\frac{2C_{f}}{mv_{x}}\left(\delta -\beta -\frac{l_{f}\gamma }{v_{x}}\right)+\frac{2C_{r}}{mv_{x}}\left(-\beta +\frac{l_{r}\gamma }{v_{x}}\right),$$

(11) $$\dot{\gamma }=\frac{2C_{f}l_{f}}{I_{z}}\left(\delta -\beta -\frac{l_{f}\gamma }{v_{x}}\right)+\frac{2C_{r}l_{r}}{I_{z}}\left(\beta +\frac{l_{r}\gamma }{v_{x}}\right).$$

For β vehicle slip angle and γ vehicle yaw rate, C_f_ is the vehicle front tire cornering stiffness, m the vehicle mass and v_x_ the longitudinal velocity. Vehicle parameters include δ as the front-steering angle, β as the body slip angle, l_f_ as the distance from center of gravity to the front axle, and v_x_ as the longitudinal velocity, C_r_ as the vehicle rear tire cornering stiffness, l_r_ as the distance between the center of gravity and the rear axle, and I_z_ as the moment of inertia for the vehicle’s yaw axis.

#### 3.1.2. Inertial Sensor

The inertial sensor determines the vehicle’s position, speed, and acceleration. It is based on a three-axis accelerometer (x,y,z) that outputs information about the vehicle’s translational speed, translational acceleration, and rotational acceleration. The inertial sensor is located in the center of the vehicle [27,62,66].

Inertial sensors, coupled with the slip angle sensor, comprise the inertial positioning system IMU (Inertial Measurement Unit), which also incorporates a three-axis accelerometer. Inertial measurements include linear acceleration, angular velocity, and angular acceleration. The dynamic parameters (roll, pitch, and yaw rate) measured by the inertial sensor are incorporated in the following relationships [94]:

(12) $$\left[\begin{array}{c} \begin{array}{c} \dot{Q_{0}}\\ \dot{q_{1}}\\ \dot{q_{2}} \end{array}\\ \dot{q_{3}} \end{array}\right]=\frac{1}{2}\cdot \left[\begin{array}{c} \begin{array}{c} 0\\ \dot{\phi }\\ \dot{\theta } \end{array}\\ \dot{\varphi } \end{array}\begin{array}{c} \begin{array}{c} \dot{-\phi }\\ 0\\ \dot{-\varphi } \end{array}\\ \dot{\theta } \end{array}\begin{array}{c} \begin{array}{c} \dot{-\theta }\\ \dot{\varphi }\\ 0 \end{array}\\ \dot{-\phi } \end{array}\begin{array}{c} \begin{array}{c} \dot{-\varphi }\\ \dot{-\theta }\\ \dot{\phi } \end{array}\\ 0 \end{array}\right]\cdot \left[\begin{array}{c} \begin{array}{c} q_{0}\\ q_{1}\\ q_{2} \end{array}\\ q_{3} \end{array}\right].$$

In three-dimensional rotation computations, the quaternion ${\left[q_{0}q_{1}q_{2}q_{3}\right]}^{T}=Q$ represents the ϕ roll, θ pitch, and ψ yaw rate. Based on the Taylor series results, the quaternion solution from instant k to moment k + 1 is

(13) $$Q\left(k+1\right)=\left(I\left(1-\frac{\Delta \Theta^{2}}{8}\right)+\frac{\Delta \Theta }{2}\right)\cdot Q\left(k\right),$$

(14) $$∆\Theta =\int_{k}^{k+1}\left[\begin{array}{c} \begin{array}{c} 0\\ \dot{\phi }\\ \dot{\theta } \end{array}\\ \dot{\varphi } \end{array}\begin{array}{c} \begin{array}{c} \dot{-\phi }\\ 0\\ \dot{-\varphi } \end{array}\\ \dot{\theta } \end{array}\begin{array}{c} \begin{array}{c} \dot{-\theta }\\ \dot{\varphi }\\ 0 \end{array}\\ \dot{-\phi } \end{array}\begin{array}{c} \begin{array}{c} \dot{-\varphi }\\ \dot{-\theta }\\ \dot{\phi } \end{array}\\ 0 \end{array}\right]dt\approx \left[\begin{array}{c} \begin{array}{c} 0\\ ∆\Theta_{\phi }\\ ∆\Theta_{\theta } \end{array}\\ ∆\Theta_{\varphi } \end{array}\begin{array}{c} \begin{array}{c} -∆\Theta_{\phi }\\ 0\\ -∆\Theta_{\varphi } \end{array}\\ ∆\Theta_{\theta } \end{array}\begin{array}{c} \begin{array}{c} -∆\Theta_{\theta }\\ ∆\Theta_{\varphi }\\ 0 \end{array}\\ -∆\Theta_{\phi } \end{array}\begin{array}{c} \begin{array}{c} -∆\Theta_{\varphi }\\ ∆\Theta_{\theta }\\ ∆\Theta_{\phi } \end{array}\\ 0 \end{array}\right].$$

For the dt sampling time, ${\Delta \theta =\left[{\Delta \theta }_{\phi }{\Delta \theta }_{\theta }{\Delta \theta }_{\varphi }\right]}^{T}$ angular increments include Δθ_ϕ_, Δθ_θ_, and Δθ_ψ_ roll, pitch, and yaw angles.

**Figure 10:**
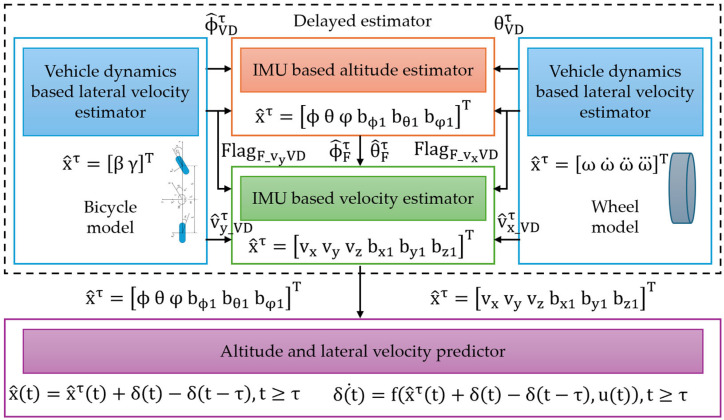
Vehicle dynamics model.

The inertial sensors IMU and VDM (vehicle dynamics model) can be used to explain the performance and safety of autonomous vehicles by properly determining the slip angle and altitude. VDM is composed of two basic parts: a delayed estimator and a predictor (Figure 10) [95]. The delayed estimator includes two types of estimators: those based on IMU data and those based on vehicle dynamics models. IMU estimators directly estimate variables like speed and attitude, but VDM estimators use mathematical models based on measurements from other sensors, like wheel speed sensors.

Under normal driving conditions, data from VDM estimators are utilized to correct errors that may develop in IMU estimates, using a Kalman filter to predict the roll and pitch angles [95].

Vehicle dynamics models provide a more in-depth understanding of the vehicle’s overall behavior, which enhances estimation accuracy. Dynamic models may become less accurate under harsh driving situations, such as hard breaking or quick bends. In such cases, the IMU estimators are temporarily separated from the VDM estimators to prevent error propagation. To synchronize the input from the two estimators, a delay is added to the VDM-based estimate. To predict the system’s current state, the predictor uses delayed estimates as well as information about the vehicle’s controls. This enables a more precise estimate of the slip angle and attitude, even in dynamic conditions [96].

#### 3.1.3. Object Sensor

Scanning the environment is an important stage for an autonomous vehicle since it offers information that allows it to perceive and understand its surroundings. This first stage is critical for obtaining a thorough and up-to-date image of the traffic situation, allowing the vehicle to make informed decisions and travel safely. To perform this comprehensive and multidimensional scanning, autonomous vehicles employ a complex network of sensors, each with particular expertise in providing information. The object sensor is a software component that simulates the operation of a real video camera in an autonomous vehicle. It detects objects in traffic and estimates their distance, with the nearest object considered the target. The data are utilized to make decisions in autonomous driving. The object sensor employs image processing techniques and AI to identify and track objects such as vehicles, pedestrians, and bicycles, and the distance between them can be calculated using trigonometric calculations [97].

Bewley et al. in [98] describe a simple and efficient technique for real-time multi-object tracking. This method focuses on the quick connection of items observed in consecutive frames, highlighting the need for accurate detection for quality tracking. Using conventional methods such as the Kalman filter and the Hungarian algorithm, the method achieves an accuracy comparable to complex systems. This method’s simplicity and efficiency make it ideal for real-time applications including pedestrian tracking in autonomous driving systems.

To predict the position of an object in the frame, a linear motion model with constant velocity is utilized, which is unaffected by other objects or camera movement.

(15) $$X={\left[u,v,s,r,\dot{u,}\dot{v},\dot{s}\right]}^{T},$$

where u and v represent the horizontal and vertical location of the target center in pixels, and s and r indicate the scale (area) and aspect ratio of the target’s bounding box, respectively [98,99].

The DPM (Deformable Part Model) algorithm, which detects surrounding vehicles and pedestrians, is an advanced object recognition method. DPM looks for and evaluates the characteristics of target objects in the images collected by the video camera, where objects are defined as a collection of parts organized in a deformable configuration. Each part represents the local attributes of an object’s appearance, whereas links between pairs of parts define the deformable configuration. The DPM algorithm learns to identify objects of interest from the background by comparing positive and negative examples. Therefore, the algorithm develops a collection of filters that respond to the object’s specific properties, such as edges, corners, and textures. A filter is built using a rectangular template defined by a matrix of dimensional vectors d. The response of the filter F at a position (x,y) on a feature map G is the “local dot product” of the filter and a sub-window of the feature map at (x,y) [100,101].

(16) $$\sum_{x^{\prime },y^{\prime }}F\left[x^{\prime },y^{\prime }\right]\cdot G\left[{x+x}^{\prime },{y+y}^{\prime }\right].$$

**Figure 11:**
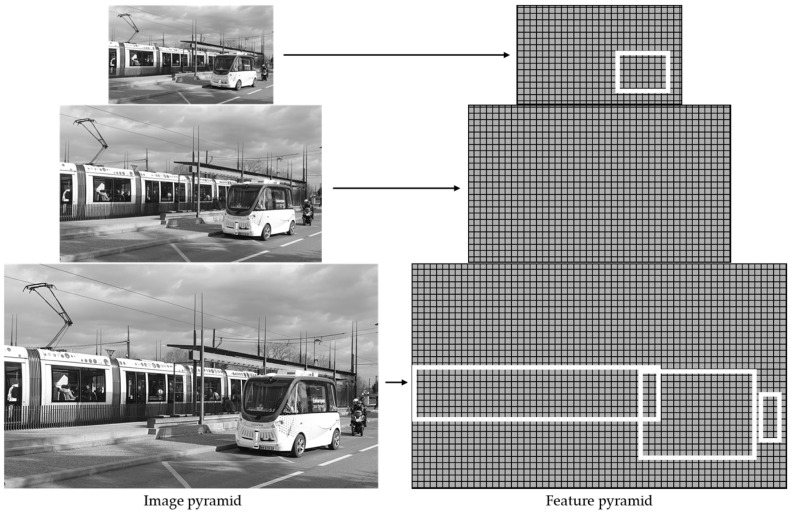
A feature pyramid undergoing instantiation of a person model within it. The part filters are positioned at double the spatial resolution of the root location.

A feature pyramid is used to specify an object’s size and position in a picture. A pyramid is a series of feature maps with varying resolutions. In practice, pyramids are created by computing a conventional image pyramid, smoothing, and repeatedly down-sampling. A feature map is then generated from each point in the picture pyramid, as depicted in Figure 11 [102,103].

In the CarMaker virtual environment, the object sensor is a virtual sensor that detects objects in traffic and calculates their distance. The most appropriate target object is determined by its proximity to the sensor [27,62]. The object sensor transmits information to the ACC system, which is responsible for automatically adapting the acceleration in the vehicle’s movement so that it maintains a consistent speed in comparison to the vehicles in front of it [67].

**Figure 12:**
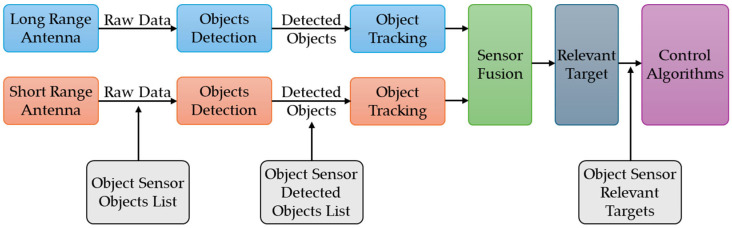
Object sensor integrated into the ACC system.

Figure 12 illustrates the structure of the ACC system, which includes the object sensor. A cluster of two sensors collects information about detected objects, one using an antenna for objects at a distance (long-range antenna) and one using an antenna for objects in close proximity to the vehicle (short-range antenna) and generates a list of intercepted objects. Raw sensor data are used to locate and track detected objects (object-sensor-detected object list), with tracking algorithms performing data fusion to ensure control over the vehicle’s cruising speed while maintaining a safe distance from the relevant detected objects [66].

The object list in the object sensor interface transmits data about the objects in the CarMaker application database that are detected by the sensor’s detection field. These objects are identified based on the characteristics of object ID (identifier), object dimensions, object orientation, distance to the object, and object speed, which correspond to the angle of incidence between the sensor beam and the object.

**Figure 13:**
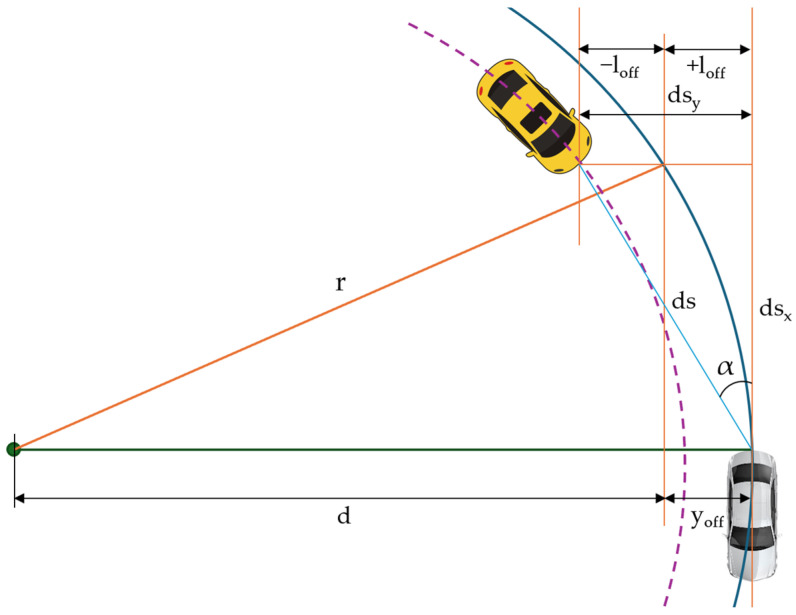
Object trajectory identification algorithm.

The algorithm for detecting the closest object is based on scanning all objects within range and selecting the relevant target objects for the object sensor by identifying the trajectory and movement lane, respectively, by calculating the movement speed and distance to the object (Figure 13) [66].

**Figure 14:**
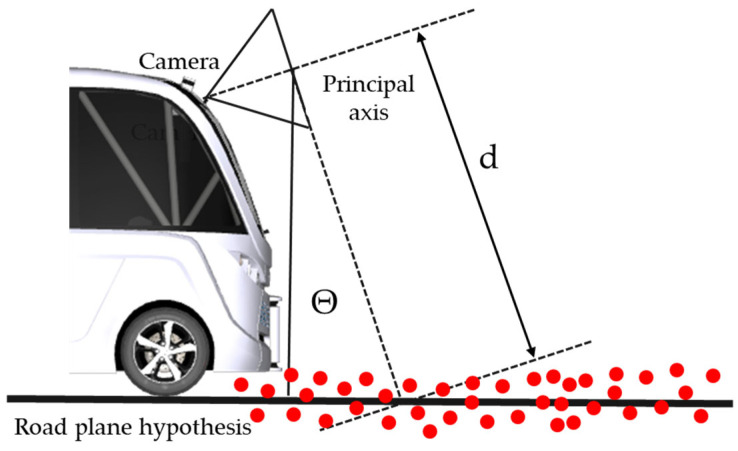
Detecting the road plane from a point cloud.

In Figure 14, ds represents the projected distance to the target vehicle, α the angle of the target vehicle in sensor frame, ds_x_ the component in the X direction of the projected distance, dsy the component in Y direction of projected distance in the sensor frame, r the turning radius, y_off_ the imaginary vehicle offset in sensor frame at the target position, l_off_ half of the vehicle lane width, ay the lateral acceleration of vehicle, and v the vehicle speed. Target selection algorithms can configure the sensor response in two modes:

- Nearest object—this is the closest visible object that is considered a relevant target;
- Nearest object in the path—this is the closest object within an interval of an estimated vehicle trajectory.

The following relationships characterize components in object sensors:

(17) $${ds}_{x}=ds\cdot \cos\left(\alpha \right),$$

(18) $${Ds}_{y}=ds\cdot \sin\left(\alpha \right).$$

The following relationships describe the vehicle’s offset in the sensor’s perception of the position of adjacent objects, and thus the limits of the vehicle’s trajectory.

(19) $$y_{off}=r-d=(r-\sqrt{r^{2}-{ds}_{x}^{2})}\cdot sign(a_{y}),$$

(20) $$L_{off}=\frac{vehicle_{lane}}{2}+lane_{offset},$$

(21) $${(y}_{off}-l_{off}<{ds}_{y})\cap {(ds}_{y}<y_{off}+l_{off}).$$

The CarMaker object sensor module generates an item list for each configured sensor, with quantities for each traffic object in the sensor’s view. After scanning the environment, the list of objects that can be recognized around the virtual vehicle model will include the following markers [66]:

- Object ID, a name or a code used to identify an object;
- Path direction (reference and closest point);
- Relative distance and velocity (between the reference and the nearest positions);
- Relative orientation in the axle x-y-z (the reference point);
- Sensor frame’s x-y-z distances (between the reference and the nearest point);
- Sensor frame’s x-y-z velocity (between the reference and the nearest point);
- Flag object has been identified (in the sensor viewing area);
- Flag object has been identified (in the observation area);
- Incidence angles between the sensor beam and the object being detected from proximity;
- Width, height, length of the object, and height above the ground.

#### 3.1.4. Free Space Sensor

The free space sensor is a software component that detects free space around the vehicle and uses this information to plan routes and avoid obstacles. The sensor creates an accurate map of the surrounding environment by combining data from several sensors, including cameras, lidar, and radar. The sensor data are analyzed to identify barriers and compute their distances. The free space sensor information is utilized to build safe and efficient trajectories, making it an important component in assuring the safety and efficiency of autonomous vehicles.

The free space sensor is an extension of the object sensor, with the sensor beam separated into horizontal and vertical segments. Each segment determines the closest point of the observed objects in traffic, in addition to the vehicle’s angle with respect to these objects and their respective speeds. The sensor scans the environment and determines the free and occupied area in the vicinity, guiding the vehicle’s progress through it [27,63].

The free space sensor plus is an extension of the sensor that detects all around items using a separate computational approach based on 3D image analysis. Three-dimensional image analysis of objects in proximity uses two filtering methods: the closest point on the object surface (nearest) and the strongest point on the object surface (strongest). The nearest finds the point on the object surface (represented by a pixel in the generated image) that is closest to the sensor position. The strongest determines the point on the object’s surface with the least reflection angle relative to the incident vector for each pixel within the sensor’s detecting range [66].

Open space identification methods use either 2D models (camera pictures) or 3D models (point clouds obtained from lidar sensors or stereo cameras). Two-dimensional approaches segment the road using low-level cues including color and texture, but they may fail if the road textures are inconsistent. However, 3D algorithms may have difficulties recognizing modest height variations, such as those between the road and the sidewalk. The hybrid method combines the benefits of 2D and 3D modeling to overcome the limits of each methodology and provide more robust open space identification [104,105,106]. Therefore, the use of 3D information obtained from the input 3D point cloud renders road plane recognition more efficient. The road plane is determined in a parametric space, which includes the plane distance from the center of the room as well as the angle between the plane normal and the room main axis. The plane in the parametric space is described by the following equation [105]:

(22) Z·sinθ−y·cosθ=d·cosθ.

This estimate suggests that the camera height and direction to the road remain constant (Figure 14). Encoders mounted on the vehicle wheel are used to correct camera and point cloud translations obtained by simultaneous localization and mapping to a metric space. The distance scale obtained from the encoders is utilized to adjust the camera translation scale, which then automatically scales the point cloud to metric space. This is required since it assists in parametrically altering the plane given a known initialization of d, dependent on the camera height [106].

#### 3.1.5. Traffic Sign Sensor

The traffic sign sensor recognizes pre-selected signs within its defined range and sight region. The sensor determines if the detected traffic sign is directly facing the vehicle and ranks the detected signs in ascending order of distance from it before identifying and classifying them. The information supplied to embedded command and control systems about detected traffic signs aids in the comprehension and interpretation of the traffic rules and conduct specified by them [27,63]. The traffic sign sensor is an ideal camera, equipped with an algorithm for recognizing traffic signs and the colors of traffic lights within its field of vision, which uses the identifiers assigned to traffic signs and traffic lights in close proximity to locate, classify, and interpret their operation [107].

**Figure 15:**
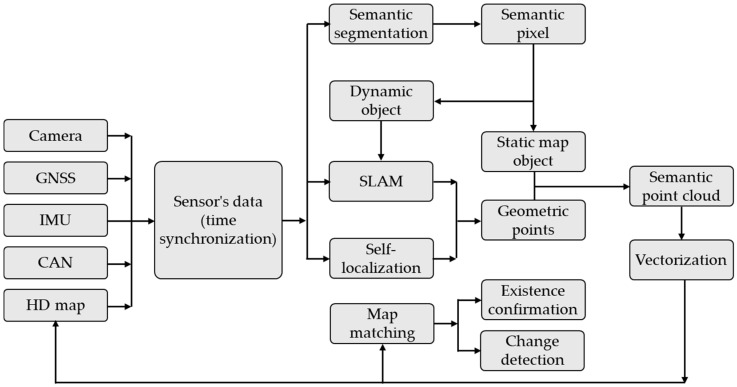
The architecture of the HD map.

HD (High-Definition) maps can provide insight into the environment in which road traffic evolves. HD maps give precise information about the environment where static road traffic occurs, including details about roads and obstacles, across a radius of more than 200 m, even in locations with no direct vision (in bends). This information, when combined with data from cameras and lidar sensors, allows for exact vehicle localization. Currently, creating HD maps requires a professional technique that includes specialized topography and mapping with an MMS (Mobile Mapping System). These maps are created by integrating road pictures with 3D data extracted from point clouds. Zhang et al. in [108] developed an architecture for real-time HD map production that includes an industrial camera, a cutting-edge GNSS (Global Navigation Satellite System)/IMU, and a high-performance computer platform on-board the vehicle (Figure 15). The semantic SLAM (Simultaneous Localization And Mapping) technology, which is based on an enhanced BiSeNet (Bilateral Segmentation Network), is used to extract semantic data from point clouds, including information about the traffic situation.

SLAM is expensive and takes a long time to create HD maps. Furthermore, HD maps may contain inconsistencies between recorded road signs and real-time local modifications. In addition to supporting drivers, intelligent object identification systems can help with roadside maintenance, including road signs, lane markings, and guardrails. Road sign recognition systems, for example, can check for potential anomalies using autonomous vehicles, as human inspection of a complete road infrastructure is difficult.

**Figure 16:**
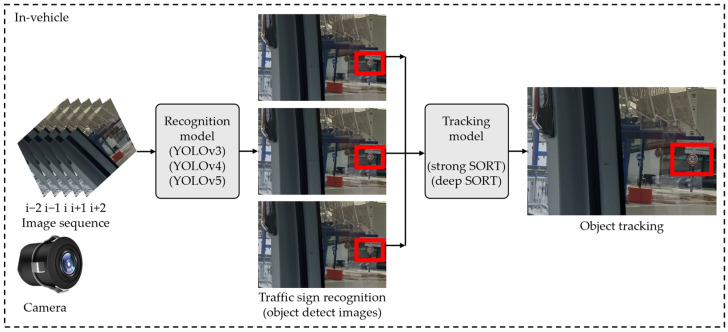
Traffic sign recognition using YOLO models.

As a result, the traffic sign recognition technique is an essential component of both autonomous driving systems and road management systems. Methods for recognizing road signs have centered on researching key aspects such as color and shape [109]. These feature-based approaches are particularly sensitive over long distances and in poor light. The usage of object detection models based on CNN has recently become popular in road sign recognition systems. DL-based object identification algorithms, such as YOLO models, aid in the correct recognition of road signs in traffic. YOLO model-based studies for road sign identification have demonstrated great performance when using publicly available reference data sets [60,110,111]. Figure 16 shows a YOLO model-based arrangement for traffic sign identification [60].

#### 3.1.6. Line Sensor

The line sensor detects road markings on the roadway in the direction of driving and organizes them into left and right lines, recording information about each. The recorded data include the lateral distance to the given reference points, the type of lines, their width and height, and the color code. To detect roadway lines, the sensor generates a sequence of planes based on seven points (five vertical planes and three inclined horizontal planes) deployed along the travel direction. Road markings on the roadway are identified by recording the intersection points of the vertical and horizontal planes created by the sensor and the lines on the road surface [66].

**Figure 17:**
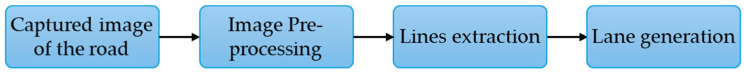
The tread detection algorithm.

**Figure 18:**
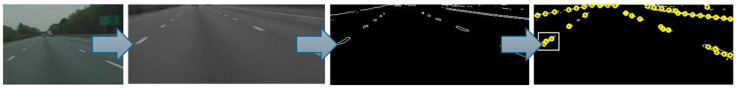
Vector direction of markings on a road.

The tread detection algorithm, illustrated in Figure 17, starts by capturing an image of the road. The image is processed in multiple phases. Initially, the image is transformed from RGB to grayscale, and then noise is reduced using a symmetric 2D Gaussian filter. The image is then processed to improve contrast in order to recognize road markings. A Sobel operator can be used to detect edges. Finally, a binary image highlighting the tread markings is generated (Figure 18) [112].

**Figure 19:**
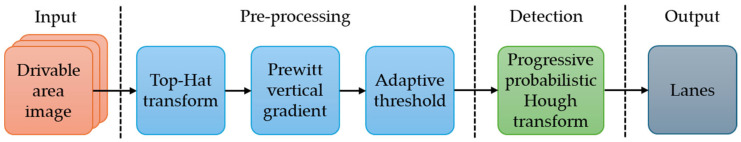
Lane marking detection algorithm.

If a combined laser scanning and video camera system is used for lane marking identification, the method is based on a top-hat transformation, which is preprocessed using the vertical-gradient Prewitt operator to generate a binary image. The binary image is then processed with a PPHT (Progressive Probabilistic Hough Transform) to detect lane markers. Figure 19 displays the lane marking detection algorithm [92].

A Top-Hat transformation is used to increase image contrast while reducing interference from non-tread marks. The Top-Hat transformation is a mathematical model that recovers small-sized bright objects and details from pictures using the following relationship:

(23) h=f−(f°b),

where f is the source image, h is the final image after performing the Top-Hat transformation, and “◦” is an operator that is realized by the Top-Hat transformation and controlled by the choice of the structuring element (b). The size of the structural element b determines how many elements are extracted from the image [92].

The Prewitt vertical-gradient operator uses the following mathematical model to detect vertical edges in an image [113]:

(24) $$\begin{array}{c} G(x,y)=|I(x+1,y-1)+I(x+1,y)+I(x+1,y+1)-I(x-1,y-1)-\\ I(x-1,y)-I(x-1,y+1)|, \end{array}$$

where I(x,y) represents the pixel intensity at the coordinates (x,y).

PPHT reduces the amount of computing required to correctly detect the markings, utilizing a linear mathematical model:

(25) y=mx+b,

where m is the slope of the line and b is the intercept at the origin.

#### 3.1.7. Road Sensor

The road sensor determines the following roadway attributes up to a specified distance: road curvature, road marker attributes (speed limits), longitudinal and lateral slope, and the distance and angle of deviation when driving. These data are sent to embedded command and control systems to perform the following functions: LK (Lane Keeping), LDW, AD (Autonomous Driving), SD (Sign Detection), EM (Energy Management), FC (Fuel Consumption), WLD (Wheel Lifting Detection), and PT (Powertrain) (Table 2) [66].

The road sensor is located in the middle of the vehicle’s front wheels. The sensor’s technique for detecting roadway features is based on projecting a point along the route reference line. The deviation represents the lateral offset of the projected point from the route reference line.

#### 3.1.8. Object-by-Line Sensor

Object-by-line sensors detect and transmit information on traffic lanes and traffic objects passing through them by assigning POI (Point Of Interest) points to each of these objects.

The route’s number of lanes is divided into LaneScope sections, which include the road axis (LaneScope Center), the left (LaneScope Left), and the right (LaneScope Right).

**Figure 20:**
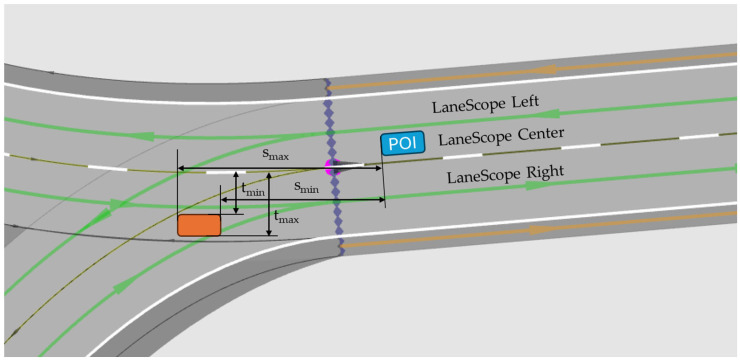
Characteristics of object-by-line sensor.

LaneScopes are used to structure information about objects and traffic lanes (Figure 20), with s_min_, s_max_, t_min_, and t_max_ defining the offsets of the extremities of traffic objects along the route [66].

The LaneScope center is calculated using the POI position. The LaneScope center course is considered to be the basis for establishing the left and right LaneScopes. The center lane course is determined by track lists generated by the computation method. The algorithm determines whether there is a lane where the POI is located. It then creates two lanes (successor/predecessor) beginning with this lane. If no acceptable successor/predecessor route is found, the relevant lane for the object-by-line sensor is terminated.

Lindenmaier et al. in [114] used a GNN (Global Nearest Neighbor) approach to assign detected objects to the reference object category in an interval d_c_, respectively, d_c,lat_, resulting in a minimal global association distance. The Mahalanobis distance relation d_MH_(xi, zj) between the reference object xi and the detection object zj defines the distance matrix D_ij_∈R^+(N,M)^, which serves as the foundation of the GNN method.

(26) $$D_{ij}=d_{MH}\left(x_{i},z_{j}\right)={\left(x_{i}^{pos}-z_{j}^{pos}\right)}^{T}\cdot S^{-1}\cdot \left(x_{i}^{pos}-z_{j}^{pos}\right),$$

where N and M are the number of reference and detected objects, and $x_{i}^{pos}$ and $z_{j}^{pos}$ are the position vectors of the respective objects. The matrix of covariance S is calculated using the cutoff distance ratio $d_{r}=d_{c}/d_{c,lat}$ as follows [115]:

(27) $$S=\left[\begin{array}{cc} \cos\alpha & -\sin\alpha \\ \sin\alpha & \cos\alpha \end{array}\right]\cdot \left[\begin{array}{cc} 1 & 0\\ 0 & d_{r}^{2} \end{array}\right]{\cdot \left[\begin{array}{cc} \cos\alpha & -\sin\alpha \\ \sin\alpha & \cos\alpha \end{array}\right]}^{T},$$

where α represents the angle of the road path at the longitudinal distance of the detected object x_i_.

### 3.2. Characteristics of Hi-Fi Sensor

#### 3.2.1. Camera Sensor

The virtual vehicle model is equipped with a sensor camera that is positioned front-to-back to provide a circular image of its surroundings. The sensor camera’s objective is to constantly monitor the movement of the virtual vehicle model along the selected route’s traffic lane in order to detect and classify static and dynamic objects in the surroundings, as well as recognize traffic signs and traffic light colors. Monocular cameras take 2D images without determining the distances to the monitored objects, whereas stereo cameras may determine the distance by measuring the difference between two images taken from various perspectives [73,116].

Environmental conditions may reduce visibility and affect the identification of nearby objects. The influence of these elements is determined using the following relationship:

(28) $$A_{Env}=max\left(1.0-\frac{RainRate}{{RainRate}_{max}},0\right)\cdot min\left(\frac{VisRangeInFog}{{VisRange}_{max}},1\right),$$

where RainRate is the rain’s intensity, and VisRangeInFog is the direct visibility under foggy conditions.

The camera sensor’s maximum error in measuring the distance (dist_Err,max_) to nearby objects will be computed using the following formula:

(29) $${dist}_{Err,max}=\frac{{dist}^{2}}{f\cdot b}\cdot d_{Err},$$

where dist is the actual distance to the monitored object, f is the focal length, and b is the baseline. d_Err_ represents the disparity error.

The x and y coordinates of the image of the item acquired by the camera are determined using the following formulas [117,118]:

(30) $$x=\frac{h\cdot (x^{\prime 2}+f^{2}-y^{\prime }\cdot f\cdot \tan\alpha )\cdot \sin\beta }{\sqrt{x^{\prime 2}+f^{2}}\cdot (f\cdot \tan\alpha +y^{\prime })},$$

(31) $$Y=\frac{h\cdot (x^{\prime 2}+f^{2}-y^{\prime }\cdot f\cdot \tan\alpha )\cdot \cos\beta }{\sqrt{x^{\prime 2}+f^{2}}\cdot (f\cdot \tan\alpha +y^{\prime })},$$

where h is the camera’s height from the ground, f is its focal length, and α is the angle between the camera’s optical axis and the horizontal line to the target. Breaking down the relationships results [119],

(32) $$\begin{array}{c} {tan}^{-1}\frac{f\cdot \tan\alpha }{\sqrt{x^{\prime 2}+f^{2}}}+{tan}^{-1}\frac{y^{\prime }}{\sqrt{x^{\prime 2}+f^{2}}}={tan}^{-1}\frac{\frac{f\cdot \tan\alpha }{\sqrt{x^{\prime 2}+f^{2}}}+\frac{y^{\prime }}{\sqrt{x^{\prime 2}+f^{2}}}}{1-\frac{f\cdot \tan\alpha }{\sqrt{x^{\prime 2}+f^{2}}}\cdot \frac{y^{\prime }}{\sqrt{x^{\prime 2}+f^{2}}}}\\ \;={tan}^{-1}\frac{\sqrt{x^{\prime 2}+f^{2}}\cdot (f\cdot \tan\alpha +y^{\prime })}{x^{\prime 2}+f^{2}-y^{\prime }\cdot f\cdot \tan\alpha }={tan}^{-1}\frac{h}{d}, \end{array}$$

(33) $$\frac{\sqrt{X^{\prime 2}+f^{2}}\cdot (f\cdot \tan\alpha +y^{\prime })}{x^{\prime 2}+f^{2}-y^{\prime }\cdot f\cdot \tan\alpha }=\frac{h}{d},$$

(34) $$d=\frac{h(x^{\prime 2}+f^{2}-y^{\prime }\cdot f\cdot \tan\alpha )}{\sqrt{x^{\prime 2}+f^{2}}\cdot (f\cdot \tan\alpha +y^{\prime })}.$$

Three-dimensional Gaussian Splatting (3DGS) is a technique that significantly enhances real-time, high-quality 3D rendering. The 3DGS method, a volume rendering technique for real-time radiance field rendering, was introduced by Kerbl et al. [120]. The 3DGS technique represents light and density in 3D space using 3D Gaussians, which are volumetric entities with color, size, and transparency. Autonomous driving is strongly reliant on an accurate and efficient representation and understanding of 3D scenes. Three-dimensional Gaussian Splatting provides substantial advantages by allowing for the real-time reconstruction of complex situations.

**Figure 21:**
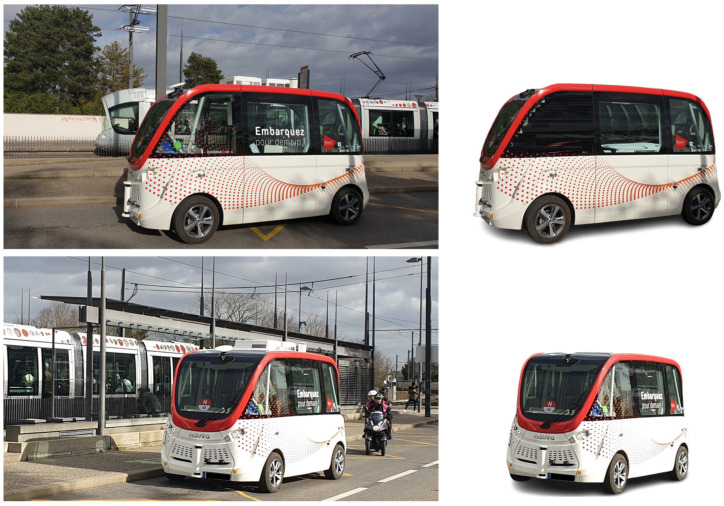
Decomposition results of Street Gaussians [121].

Important tasks including obstacle identification and path planning depend heavily on spatial information processing. Street Gaussians, for example, model scenes as 3D Gaussian point clouds, capturing both geometry and appearance, and enable the separation of static backgrounds from dynamic foreground elements, which is critical for semantic segmentation in autonomous systems (Figure 21) [121].

Thus, the process is analogous to triangle rasterization in computer graphics, except instead of triangles, it uses 3D Gaussians. This approach enables the representation of complex situations while maintaining a balance between rendering efficiency and visual fidelity [122]. The 3DGS algorithm represents a scene using a collection of 3D Gaussians [121].

Each Gaussian is specified by parameters including transparency, location, and shape, which are tuned to represent the scene from multiple perspectives. To render an image, 3DGS organizes the Gaussians according to their distance from the camera and then mixes them using alpha blending. This approach allows for the production of smooth and realistic visuals in real time. The 3D Gaussian representation can be stated mathematically as follows [121,122]:

(35) $$L_{3DGS(x,y,z,\theta ,\phi )}=\sum_{i}G(x,y,z,\mu_{i},\Sigma_{i})\cdot c_{i}(\theta ,\phi ),$$

where G is the Gaussian function with mean μ_i_ and covariance Σ_i_, and c is the view-dependent color.

**Figure 22:**
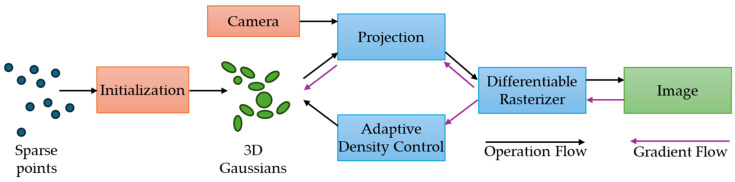
Three-dimensional Gaussian Splatting Process: from sparse point cloud initialization and adaptive density optimization to efficient training via tile-based rendering [123].

Figure 22 illustrates the process from the initial sparse point cloud to the final image creation, with essential steps including initialization, 3D Gaussian representation, projection, rasterization, and adaptive density control [123].

The 3DGS technique has various advantages over conventional 3D cameras and rendering systems. It offers a more efficient and scalable rendering mechanism, enabling real-time rendering while preserving great visual quality. The 3DGS method also enables simple scene manipulation and editing, making it a useful tool for a variety of applications.

#### 3.2.2. Global Navigation Sensor

The global navigation sensor locates a vehicle by using the positions of at least four geostationary GPS satellites. The sensor determines real-time positioning in geographic coordinates using information about the transmission time of the extrapolation signal emitted by the satellites and received by the vehicle’s receiver (x,y,z,t) [124].

CarMaker can represent any position of the virtual vehicle model in the global road architecture as a geographic point on the Earth’s surface. This uses the GCS (Geographic Coordinate System) coordinate system, which consists of latitude, longitude, and altitude.

**Figure 23:**
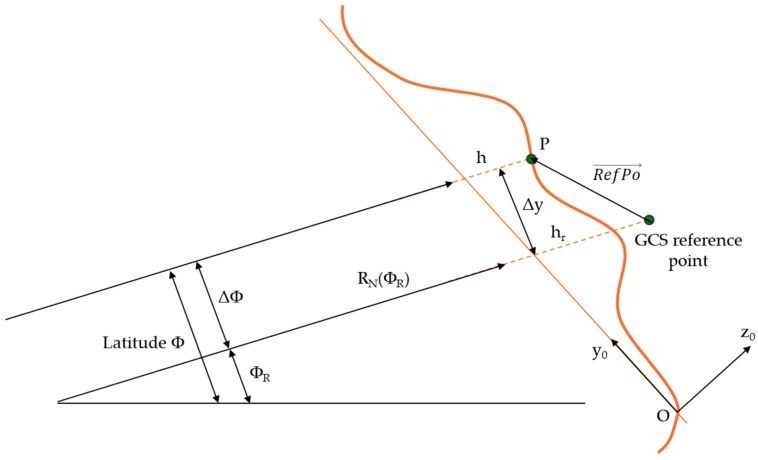
Calculation of the latitude for the global navigation sensor.

The origin of the road frame on the Earth’s surface is determined using the FlatEarth projection method using GCS reference points (Figure 23). This projection method ignores the Earth’s curvature around the GCS reference point when calculating the relative position $\vec{RefPo}$ of point P in the road frame. The elevation value h at point P is calculated as follows [66]:

(36) $$h=h_{R}+\vec{RefPo}{\left[z\right]}_{0}.$$

The relative latitude Δϕ can be calculated as follows:

(37) $$\Delta \Phi =asin\left(\frac{\Delta y}{R_{N}\left(\phi_{R}\right)+h}\right)\approx \frac{\Delta y}{R_{N}\left(\phi_{R}\right)+h}=\frac{\vec{RefPo}{\left[y\right]}_{0}}{R_{N}\left(\phi_{R}\right)+h},$$

where $R_{N}\left(\phi_{R}\right)$ is the radius of the Earth’s ellipsoid in the north, which is determined by the latitude of the GCS reference point. The latitude ϕ of point P is transformed into

(38) $$\Phi =\phi_{R}+\Delta \phi .$$

Similarly, the longitude at point P can be determined using the following formula:

(39) $$\lambda =\lambda_{R}+\frac{\vec{RefPo}{\left[zx\right]}_{0}}{\left(R_{E}\left(\phi_{R}\right)\cdot \cos\left(\varphi_{R}\right)\right)},$$

where $R_{E}\left(\phi_{R}\right)$ is the radius of the Earth’s ellipsoid in the east, which is determined by the latitude of the GCS reference point. The factor cos(ϕ_R_) considers decreasing radius with increasing latitude.

D-GNSS (Differential-Global Navigation Satellite System) with differential RTK (Real-Time Kinematic) correction reduces vehicle positioning errors by applying a differential correction relative to the coordinates of a reference base station, which then uses signals transmitted by satellites. The delivery of D-GNSS differential corrections with RTK from the reference base station to the vehicle takes place via mobile data connections [27].

CarMaker’s virtual car model employs Cartesian coordinates (x, y, z), geocentric coordinates ECEF (Earth-Centered, Earth-Fixed), and ellipsoidal coordinates (ϕ latitude, λ longitude, h elevation) for geostationary satellite positioning. GDOP (Geometric Dilution of Precision) refers to the accuracy of the computed position of the vehicle receiver in reference to the geostationary position of visible satellites.

#### 3.2.3. Radar Sensor

The radar sensor detects static and dynamic objects on the virtual vehicle route using the SNR signal. The sensor detects things using cellular units and accounts for the effects of overlapping traffic items. The detected items are identified using particular RCS (Radar Cross-Section) cross-section maps that take into account the angle of incidence and the signal reflected by the traffic objects. Depending on the signal-to-noise ratio, surrounding objects will be recognized, removed, or regarded as false negatives [27].

RCS is defined as the cross-section of a detected object that intercepts the most amount of power transmitted by the radar sensor. It is determined by the following parameters: the size and shape of the object, the antenna orientation angle, the frequency and polarization of the radar waves, the object’s electromagnetic properties, and the surface structure. Radar sensor simulation is complex due to the dispersion effect of radar waves in virtual settings, and it is achieved using physically interpretable characteristics such as the distance to nearby objects, the movement speeds of traffic items, and their angular locations.

Elster et al. in [125] used the DVM (Double Validation Metric) methodology to validate radar sensor data virtually. DVM uses the reliability and repeatability of radar sensor readings to effectively quantify deviations between distributions for various types of detected objects. The measurement data (M1, M2) are preprocessed and filtered, and the number of points resulting from the measurements is determined for each data set using the EDF (Empirical Cumulative Distribution Function) methodology.

**Figure 24:**
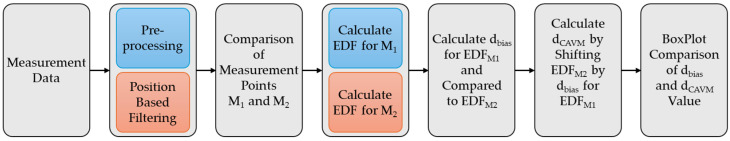
DVM methodology.

The deviation of mean values d_bias_ for measurement data M1 is calculated and compared to that of measurement data M2. The shape deviation of the distribution function d_CAVM_ is calculated to highlight the difference in signal scattering between M1 and M2 (Figure 24) [126].

The radar equation defines the physical relationships connected to the features of the radar sensor, resulting in the received signal power ρ_s_(r_s_,υ_s_,ϕ_s_) [66,127,128].

(40) $${\left|\varrho_{sP}\left(r_{s},\upsilon_{s},\phi_{s}\right)\right|}^{2}=\frac{P_{Rx}}{P_{Tx}}=\frac{\lambda_{c}^{2}\cdot G_{Tx}\left(\upsilon_{s},\phi_{s}\right)\cdot G_{Rx}\left(\upsilon_{s},\phi_{s}\right)\cdot \sigma_{s}}{{\left(4\pi \right)}^{3}\cdot r_{s}^{4}}\cdot F{\left(r_{s},\upsilon_{s},\phi_{s}\right)}^{4},$$

where ρ_s_ is the impulse response for s point scatterer, positioned at r_s_ distance at an angular position, for υ_s_ elevation and ϕ_s_ azimuth, λ_c_ is the wavelength of the carrier frequency, G_Tx_ is the gain of the transmitter antenna, G_Rx_ is the gain of the receiver antenna, and σ_s_ is the reflection coefficient of the point scatterer.

A gain map is used to highlight antenna features, which is characterized by a unidirectional gain factor parameterized by azimuth and elevation in the scanning direction. The following equations describe field strength and antenna gain [129]:

(41) $$f\left(\theta ,\phi \right)=\frac{\sin\left(\pi \frac{a}{\lambda }\sin\theta \cos\phi \right)}{\left(\pi v_{y}\right)}\frac{\sin\left(\pi \frac{b}{\lambda }\sin\theta \sin\phi \right)}{\left(\pi v_{z}\right)},$$

The aperture dimensions include θ elevation, ϕ azimuth, and a and b major lobes.

The detection threshold can be determined by the lowest detectable value of SNR_min_ and is calculated using the parameters of the minimal probability of detection P_Dmin_ and the probability of false alarm P_FA_ [66]:

(42) $${SNR}_{min}=2{\left({erfc}^{-1}\left(2P_{FA}\right)-{erfc}^{-1}\left(2P_{min}\right)\right)}^{2}.$$

The strength S of the received signal is determined by the radar equation:

(43) $$S=\frac{PG^{2}\lambda^{2}RCS}{{\left(4\pi \right)}^{3}r^{4}}\cdot \frac{1}{L_{A}L_{atm}},$$

where P is the transmitted power, G is the gain of the antenna, λ is the wavelength, r is the distance from the radar sensor to the object, L_A_ is the additional system loss, and L_atm_ is the atmospheric loss.

**Figure 25:**
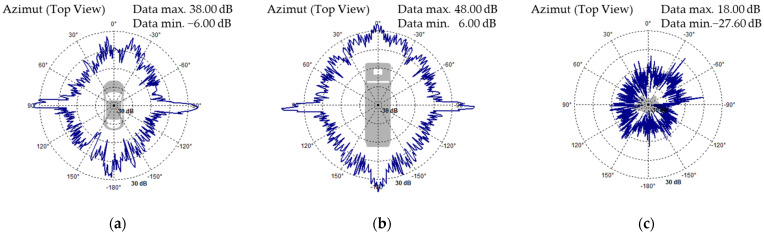
RCS of the various objects: (**a**) vehicle, (**b**) truck, (**c**) pedestrian.

The specific RCS cross-section maps for close objects are determined by radar sensor resolution, object size, direction of incidence, object occlusion, and object merging (Figure 25) [27].

**Figure 26:**
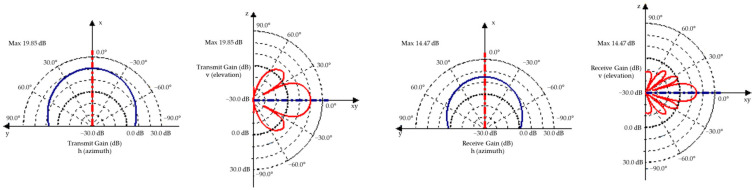
Transmit/receive (azimuth/elevation) gain map.

The transmit gain map adjusts the power of the delivered signal to 3D objects. The transmit gain is calculated using linear interpolation of the parameterized gain map (Figure 26).

### 3.3. Characteristics of RSI Sensor

#### 3.3.1. Lidar RSI Sensor

The lidar (light detection and ranging) sensor operates based on measuring the ToF for a beam of laser pointers with a wavelength of 905 nm that is emitted at objects in the area and computing the reception time of this beam reflected by these items. Lidars, including RSI sensors, generally use the ToF principle to determine distance. This involves generating a laser pulse and measuring its return time after hitting an object [130].

The lidar 2D sensor captures information about nearby objects by sending a single laser beam onto a revolving mirror perpendicular to the axis of rotation. The lidar 3D sensor gathers information about nearby objects by shooting a beam of laser rays through a rotating mechanism, resulting in a point cloud for the contour of these objects and the capacity to build high-precision 3D maps.

Lidar RSI sensors use a rotating equipment to guide a laser beam across their range of vision. This scanning is often accomplished using rotating polygonal mirrors that direct the beam with each facet, resulting in a balance of accuracy and speed. Alternatively, revolving prisms can refract the beam with great precision and stability. A recent technique uses MEMS (Micro-Electro-Mechanical Systems) technology, in which small mirrors direct the beam, allowing for more compact designs but potentially limiting the range and scan angle.

Lidar generates a 3D representation of the viewed reality by measuring the ToF at multiple points within its FoV (field of view). The set of points is referred to as a point cloud. The nth measurement point (p_n_) in the lidar reference system {L} can be expressed as follows [131]:

(44) $${{\left\{L\right\}}_{p}}_{n}={\left[{{\left\{L\right\}}_{x}}_{n},{{\left\{L\right\}}_{y}}_{n},{{\left\{L\right\}}_{z}}_{n}\right]}^{T}=\frac{c}{2}{t_{ToF,}}_{n}\cdot {{\left\{L\right\}}_{\hat{s}}}_{n},$$

where c is the speed of light in air, t_ToF,n_ is the ToF measure, and ŝ_n_ is the unitary vector indicating the scanning direction of the lidar in its reference system {L}. Equation (43) shows that accurate and exact point clouds require both the ToF measurement (t_ToF,n_) and the scanning direction (ŝ_n_). The scanning direction of the lidar, designated as ŝ, is expressed in the laser source’s reference system {I}. The mirror’s tilt angles (α and β) correspond to horizontal and vertical directions, respectively. This relationship is further developed in the following equations [131]:

(45) $${\left\{I\right\}}_{\hat{s}}={\left\{I\right\}}_{\hat{i}}-\left(\gamma (\alpha ,\beta ,\varphi \right)-\sqrt{{\left(\gamma (\alpha ,\beta ,\varphi \right)}^{2}})\cdot {\left\{I\right\}}_{\hat{n}},$$

(46) $${\left\{I\right\}}_{\hat{n}}=\left[\begin{array}{c} sin\alpha \cdot cos\beta \\ cos\varphi \cdot sin\beta +sin\varphi \cdot cos\alpha \cdot cos\beta \\ sin\varphi \cdot sin\beta -cos\varphi \cdot cos\alpha \cdot cos\beta \end{array}\right],$$

(47) $$\gamma \left(\alpha ,\beta ,\varphi \right)={\left\{I\right\}}_{\hat{n3}}=sin\varphi \cdot sin\beta -cos\varphi \cdot cos\alpha \cdot cos\beta .$$

In the laser source reference system {I}, γ represents the third component of the normal vector $\hat{n}$, whereas α and β represent the scanning tilt angles, direction, and mirror surface. Each point in the cloud is described by its (x, y, z) coordinates, which are computed from the measured distance, the horizontal angle (determined by the revolving mirror or prism), and the vertical angle (typically fixed but variable in multi-emitter systems).

For the CarMaker virtual vehicle model, lidar 2D and 3D sensors are defined as lidar RSI sensors that provide information about nearby objects and behave just like genuine sensors. The calibration of the lidar virtual sensor was performed by using simulation data from various surroundings that were correlated with the motion sensor [132].

**Figure 27:**
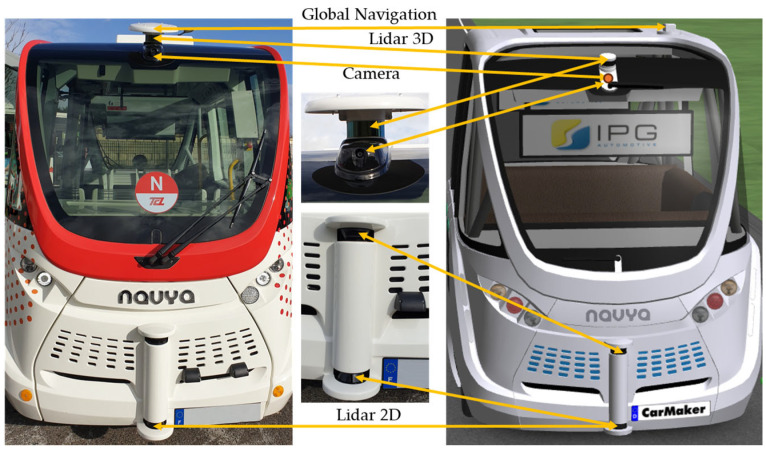
RSI sensor distribution on the virtual vehicle model’s body structure.

Lidar 2D sensors are used in pairs of two units to reduce coverage gaps and ensure continuous visibility throughout the entire surface of a horizontal scanning plane (P2D) with a viewing angle of 180° and a field of view centered on the virtual vehicle model’s median longitudinal plane. Lidar 3D sensors scan on 16 channels, following a plane (P3D) with successive inclinations along an increasing angular axis with respect to the upper horizontal scanning plane. They have a viewing angle of 30° and an opening of ±15° with respect to the median longitudinal plane of the virtual vehicle model (Figure 27).

The interaction modes of the lidar RSI sensor are classified using the following criteria [65]:

1. Uniformly distributed diffuse reflected laser rays, regardless of the direction of the incident ray (Lambertian reflection), with the intensity of the reflected ray decreased by the inverse of the angle between the incident ray and the normal of the reflective surface;
2. Retroreflective, meaning that incident laser rays are reflected back in the direction of the emitter, with the intensity of the reflected ray being reduced based on the reflectance parameters and incident angle;
3. Specular, meaning that the incident and reflected laser rays form identical angles with the normal of the reflective surface, and the incident and reflected laser rays are in the identical plane;
4. Transmissive, meaning that the incident laser photons keep their course but are attenuated by transmission.

#### 3.3.2. Ultrasonic RSI Sensor

**Figure 28:**
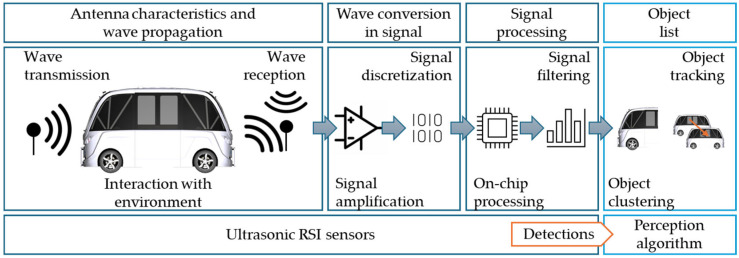
Signal chain of the ultrasonic RSI sensor models.

CarMaker’s ultrasonic RSI model is based on the signal chain of a real ultrasonic sensor. The process begins with a transmitter illuminating the environment and emitting sound waves (Figure 28). This method divides sound waves into a finite number of rays. This provides for a balance between simulation speed and physical accuracy. Environmental circumstances with physical effects on wave propagation are considered, and the Helmholtz equation is used to compute electrical scatter fields on object surfaces utilizing parameterizable material properties to predict reflections. Every suitable ray returns to the receiver [133].

**Figure 29:**
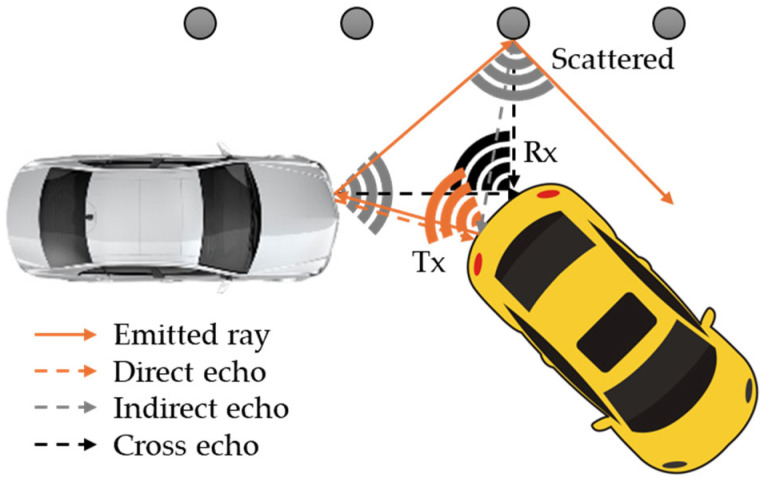
A diagram of the ray tracing algorithm used to simulate a sound wave.

For all detections, the sensor model returns the sound pressure level as well as the flight time. It accounts for dense packaging and associated effects by optionally sensing cross-echoes between sensors. Interfaces enable the replacement of individual steps with a user-defined code, and clustering on available GPUs improves performance [133], as seen in Figure 29.

**Figure 30:**
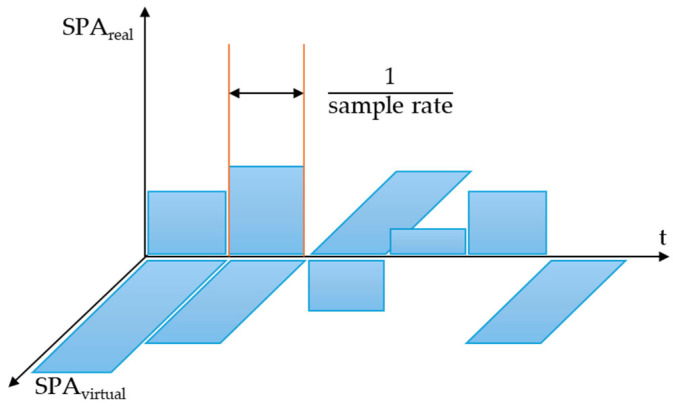
SPA full wave form.

The ultrasonic RSI sensor uses mechanical acoustic pressure waves that are reflected by obstacles in the immediate vicinity of the virtual vehicle model using the ToF principle, and the distance to the respective obstacles is accurately calculated using the SPA (Sound Pressure Amplitude) (Figure 30). The sensor considers the impacts of overlapping objects in the area, the effects of physical propagation, and the classification as false positives or false negatives of the observed objects. The propagation modes of acoustic pressure waves are classified using the following criteria [66]:

1. Direct Echo: The acoustic pressure wave is reflected once by an object in close proximity and received by the emitting sensor.
2. Indirect Echo: The acoustic pressure wave is reflected at least twice by objects or surfaces in the vicinity and received by the emitting sensor.
3. Repeated Echo: The emitting sensor receives the acoustic pressure wave after it has been reflected by nearby objects or surfaces.
4. Cross Echo: The reflected acoustic pressure wave is received by a sensor other than the originating sensor, resulting in a propagation mode known as cross echo.
5. Road Clutter: This occurs when the acoustic pressure wave is reflected by bumps or irregularities in the roadway.

Ultrasonic RSI sensors are installed in the vehicle’s front bumper, side panels, and rear bumper.

## 4. Virtual Sensor Parametrization

### 4.1. Ideal Sensor Parametrization

The ideal sensors incorporated in the virtual vehicle model are as follows: slip angle, inertial, object, free space, traffic sign, line, road, and object by line.

To highlight the major parameters generated by the indicated ideal sensors, a series of simulations have been performed on the proposed route (see Figure 8), digitized, and implemented in the IPGRoad/CarMaker application.

**Figure 31:**
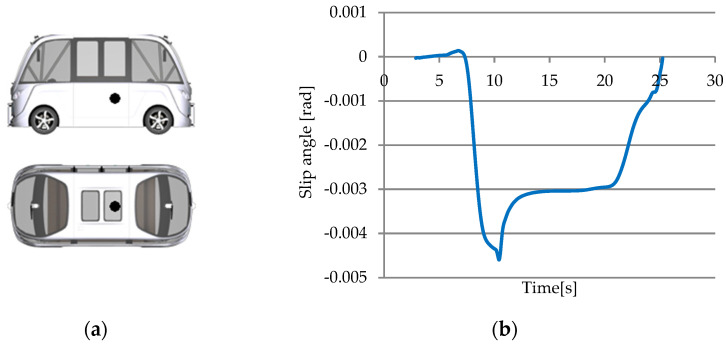
Slip angle sensor parameterization and generated parameter. (**a**) Sensor position (**b**) Sensor parameter.

For the slip angle sensor (Figure 31a), one parameter has been chosen: Sensor.SAngle.SL00.Ang (Figure 31b). The analysis was performed on an area of the route traveled, starting with a straight road section, then a left turn and another straight road section.

The graphic illustrates how the slip angle sensor’s values change when the vehicle approaches a left curve (second 7) and returns to a straight road segment (second 25). According to the data displayed on the graph in the image, the maximum slip angle is around 0.0045 radians (0.25 degrees).

**Figure 32:**
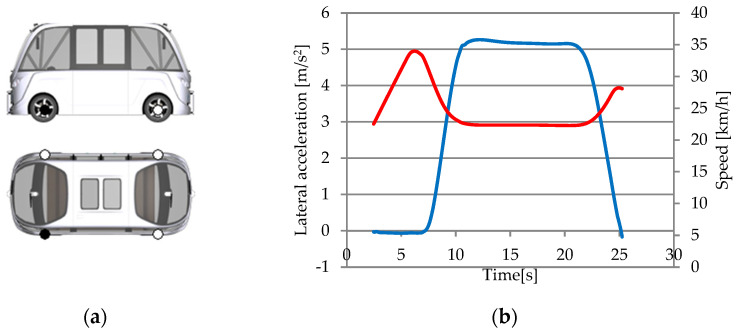
Inertial sensor parameterization and generated parameters. (**a**) Sensor position (**b**) Sensor parameters.

For the inertial sensor (Figure 32a), two parameters have been chosen: Sensor.Inertial.YRS.Vel_0.x (red curve) for the speed in the x direction and Sensor.Inertial.YRS.Acc_B.y (blue curve) for the lateral acceleration (Figure 32b).

The graphic shows that lateral acceleration increases from 0 m/s^2^ when moving straight to 5.3 m/s^2^ when turning. The vehicle’s lateral acceleration reduces as it exits the turn, returning to near-zero m/s^2^ levels. The sensor’s observed lateral accelerations correspond with typical turns.

**Figure 33:**
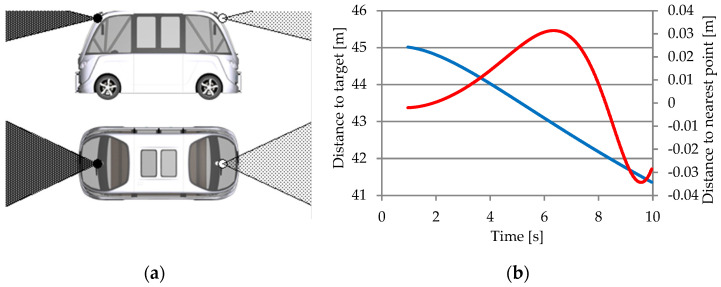
Object sensor parameterization and generated parameters. (**a**) Sensor position (**b**) Sensor parameters.

For the object sensor (Figure 33a), two parameters have been chosen: Sensor.Object.RadarL.relvTgt.NearPnt.ds.x (red curve) represents the distance (ds) along the specified x-axis, corresponding to the direction of the sensor system axes, from the most relevant target (relvTgt), respectively, the detected object that is most important to track; Sensor.Object.RadarL.relvTgt.NearPnt.ds.y (blue curve) represents the distance (ds) along a specific y-axis parallel to the sensor system axes and the detected object’s nearest point (NearPnt) (Figure 33b). This value represents the shortest distance between the sensor and the relevant target (a point on the target), which could be a vehicle, a pedestrian, etc.

The graphic shows the variations in x and y coordinates over time for the nearest point on an obstacle detected by the sensor. The coordinates corresponding to the z-axis (height) have not been shown since they contain relatively minor fluctuations that have no significant effect on the position of the obstacle.

**Figure 34:**
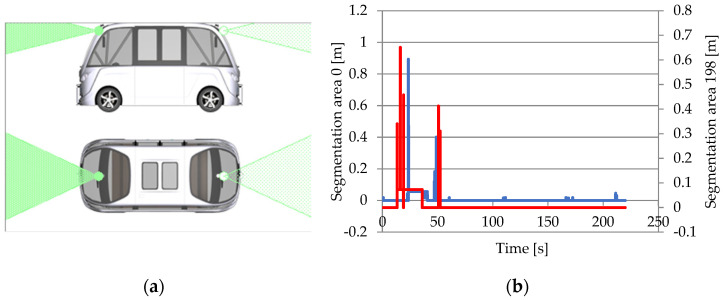
Free space sensor parameterization and generated parameters. (**a**) Sensor position (**b**) Sensor parameters.

For the free space sensor (Figure 34a), two parameters have been chosen—Sensor.FSpace.Front.Segm.0.ds.x (red curve), and Sensor.FSpace.Front.Segm.198.ds.x (blue curve)—which represent the segmentation areas, includes the closest point of a detected traffic obstacle. The segmentation area is divided into four quadrants. Figure 34b shows how the various parameters change along the x-axis, with each parameter representing a quadrant of the segmentation area. The graphs illustrate the change in expected parameter values across the segmentation region over the same time frame. Analyzing the graph which corresponds to the parameter Sensor.FSpace.Front.Segm.198.ds.x, one can see no more variations in the specified parameter after second 52, indicating that no obstacle was identified in the associated quadrant.

**Figure 35:**
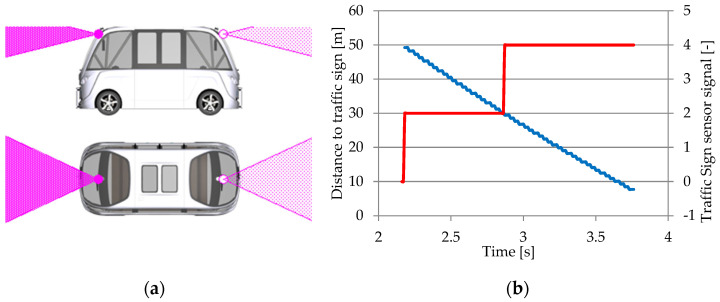
Traffic Sign sensor parameterization and generated parameters. (**a**) Sensor position (**b**) Sensor parameters.

For the traffic sign sensor (Figure 35a), two parameters have been chosen: Sensor.TSign.FrontCam.SpeedLimit.0.ds.x (blue curve) indicates the distance between the detected indicator (Speed Limit sign) and the direction of travel (x), respectively; Sensor.TSign.FrontCam.SpeedLimit.nSign (red curve) indicates the signal generated by the sensor when it detects a speed limitation sign (Figure 35b). When the distance between the vehicle (sensor) and the road sign is less than 30 m, the signal reaches its maximum value.

**Figure 36:**
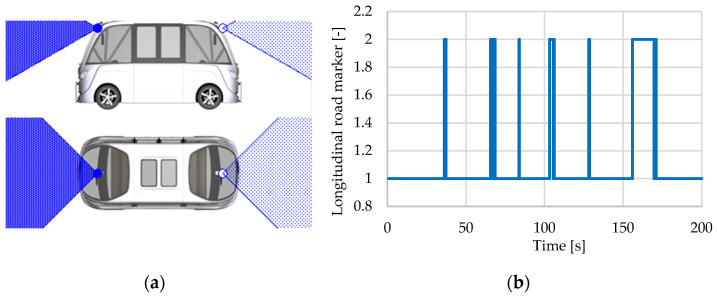
Line sensor parameterization and generated parameter. (**a**) Sensor position (**b**) Sensor parameters.

For the line sensor (Figure 36a), one parameter has been chosen: Sensor.Line.Front.RLines.1.Type (Figure 36b) indicates the type of longitudinal road marking located on the right side of the road in the virtual vehicle model’s direction of travel. The graph shows a variation in the signal of the parameter in the range of 1 to 2. The parameter’s value of 1 indicates the presence of a longitudinal road marking made up of a simple dashed line, while the parameter’s value of 2 indicates the presence of a longitudinal road marking made up of a single continuous line.

Changing the parameter value from 1 to 2 moves the virtual vehicle model from a road section with a simple dashed line (1) to a road section with a continuous line (2). If the parameter value is constant (1 or 2), the type of longitudinal road marking does not differ; if the values fluctuate frequently, a road section with many changes in longitudinal road marking will result.

**Figure 37:**
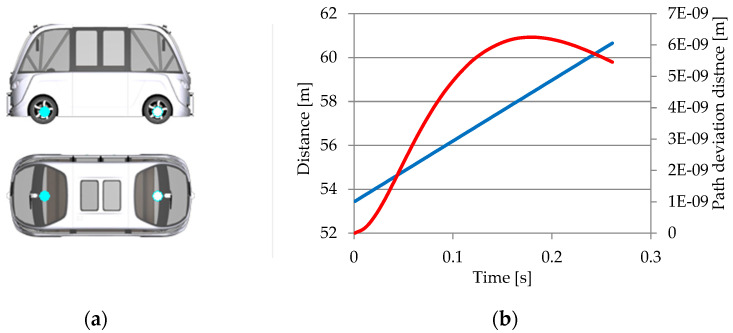
Road sensor parameterization and generated parameters. (**a**) Sensor position (**b**) Sensor parameters.

Two parameters have been chosen for the road sensor (Figure 37a): Sensor.Road.AlongR.Path.tx (blue curve) represents the vehicle displacement along the x-axis, and Sensor.Road.AlongR.Path.DevDist (red curve) represents the deviation from the planned route (Figure 37b). Over a route length of around 8 m, the deviation ranges between 0 and 7 × 10^−9^ m.

**Figure 38:**
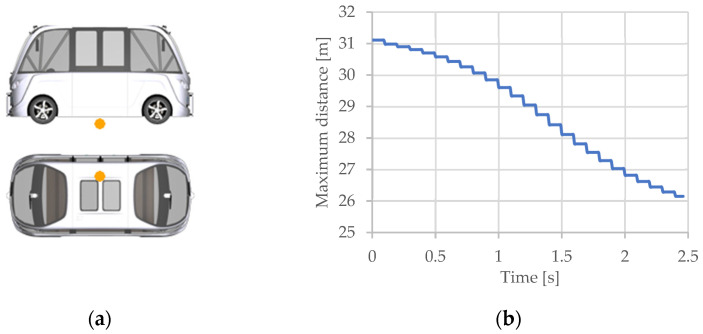
Object-by-line sensor parameterization and generated parameter. (**a**) Sensor position (**b**) Sensor parameter.

For the object-by-line sensor (Figure 38a), one parameter has been chosen: Sensor.ObjByLine.OBL00.LinesC.0.ObjF.0.sMax (Figure 38b) highlights the variation in maximum distance between the virtual vehicle model and the POI over a 2.5 s period. Figure 21 shows that S_max_ decreases over time, indicating that the vehicle has approached the POI, where LinesC indicates the LaneScope Center section.

### 4.2. Hi-Fi Sensor Parametrization

The Hi-Fi sensors incorporated in the virtual vehicle model are as follows: (a) camera, (b) global navigation, (c) radar.

To highlight the major parameters generated by the indicated Hi-Fi sensors, a series of simulations have been performed on the proposed route (see Figure 8), digitized, and implemented in the IPGRoad/CarMaker application.

**Figure 39:**
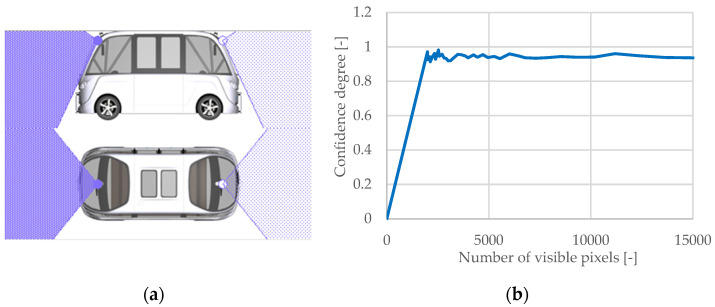
Camera sensor parameterization and generated parameter. (**a**) Sensor position (**b**) Sensor parameter.

For the camera sensor (Figure 39a), two parameters have been chosen: Sensor.Camera.CA00.Obj.0.nVisPixels (x-axis) indicates the number of visible pixels of the object recognized by the sensor, and Sensor.Camera.CA00.Obj.0.Confidence (y-axis) indicates the confidence degree of object detection (Figure 39b). The graph illustrates the efficacy of object detection based on their size in the image. If an object has many visible pixels but a low confidence level, the recognition algorithm becomes unreliable. The maximum confidence level for recognized items is 1.

**Figure 40:**
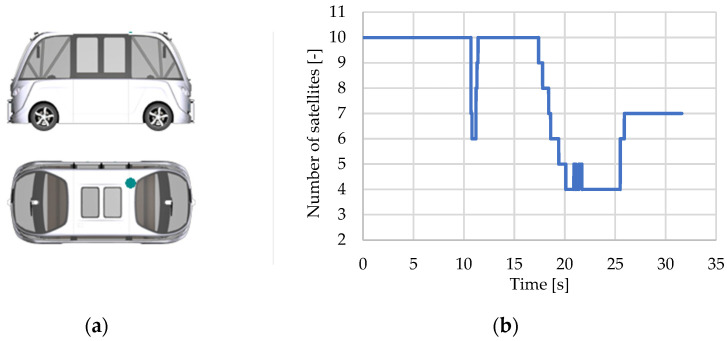
Global navigation sensor parameterization and generated parameter. (**a**) Sensor position (**b**) Sensor parameter.

For the global navigation sensor (Figure 40a), one parameter has been chosen, Sensor.GNav.Receiver.SatNoDirectView, that highlights the number of satellites that are directly visible to the GNSS receiver in a specific time interval (Figure 40b).

The satellites’ direct visibility influences the measurement accuracy. The maximum number of visible satellites ranges from 4 to 10 and is influenced by tall buildings or underground passages that the vehicle passes through, and by extreme weather conditions.

**Figure 41:**
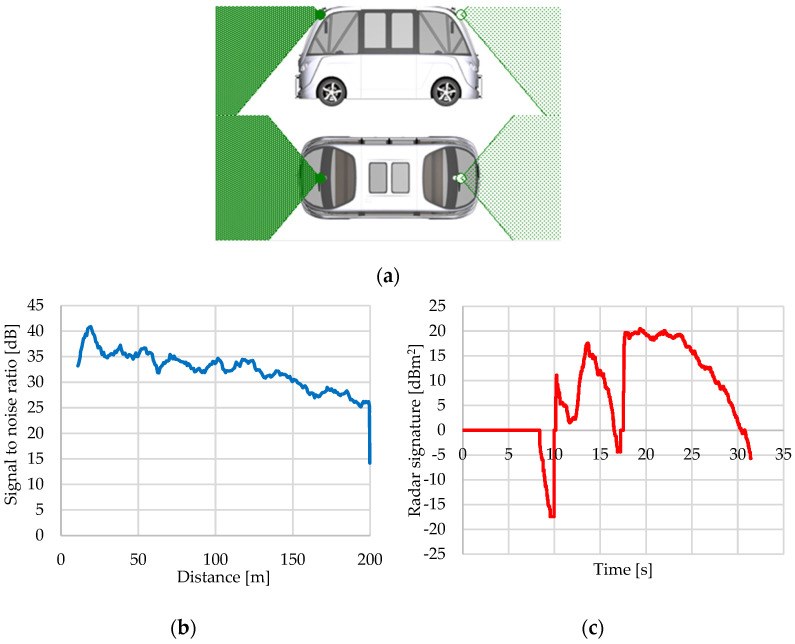
Radar sensor parameterization and generated parameters. (**a**) Sensor position (**b**) Sensor parameter signal to noise ratio (**c**) Sensor parameter radar signature.

For the radar sensor (Figure 41a), two parameters have been chosen: Sensor.Radar.RA00.Obj0.Dist (blue curve) represents the variation in the signal-to-noise ratio (SNR) as a function of the distance from the object, and Sensor.Radar.RA00.Obj0.SNR represents the signal to noise ratio (Figure 41b). The graph illustrates the radar signal’s detection efficiency versus background noise as a function of distance from the target object. A higher signal-to-noise ratio suggests better detection. As the object moves away, the signal-to-noise ratio decreases, resulting in poorer detection.

Another parameter evaluated for the radar sensor is Sensor.Radar.RA00.Obj0.RCS (red curve), which represents a variation in the RCS parameter (target object’s radar signature) in the case of detection (Figure 41c). The graph shows significant changes in RCS parameter values, ranging from −18 dBm^2^ to 21 dBm^2^. The analysis of the RCS parameter indicates that the radar sensor detects obstacles in the virtual vehicle model’s travel environment, ranging from small (negative values for poor detection) to large (positive values for good detection).

### 4.3. RSI Sensor Parametrization

The RSI sensors incorporated in the virtual vehicle model are as follows: (a) lidar RSI, (b) ultrasonic RSI.

To highlight the major parameters generated by the indicated RSI sensors, a series of simulations have been performed on the proposed route (Figure 8), digitized, and implemented in the IPGRoad/CarMaker application.

**Figure 42:**
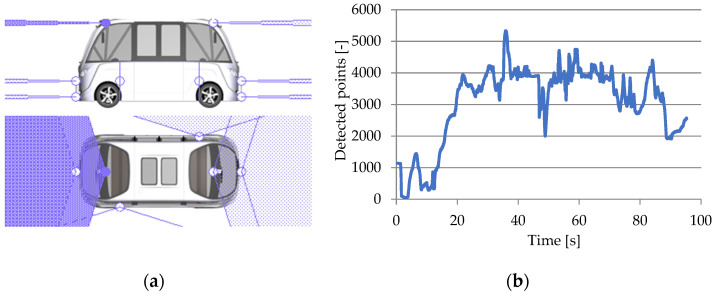
Lidar RSI sensor parameterization and generated parameter. (**a**) Sensor position (**b**) Sensor parameter.

For the lidar RSI sensor (Figure 42a), one parameter was chosen: Sensor.LidarRSI.LIRS00.nScanPoints indicates the number of points detected in an interval of time (Figure 42b). The graph’s parameter variations allow for an investigation of scanning conditions in particular areas of the virtual vehicle model’s travel path. A high parameter value results in a congested area with many detected obstacles, whereas a low parameter value results in an area with fewer detected obstacles.

**Figure 43:**
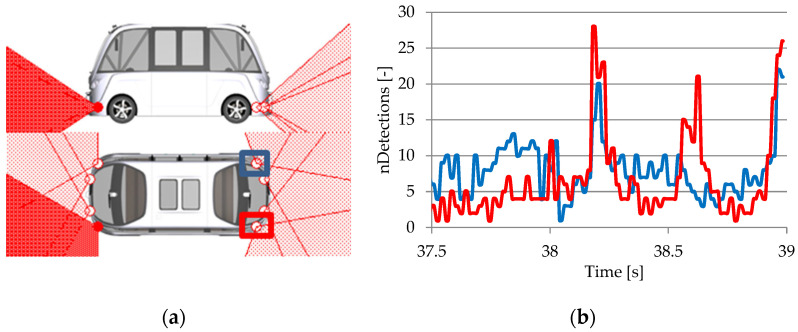
Ultrasonic RSI sensor parameterization and generated parameters. (**a**) Sensor position (**b**) Sensor parameters.

The ultrasonic RSI sensor (Figure 43a) shows the variation in a specified parameter for two identical sensors: Sensor.USonicRSI.Tx.USRS00.Rx.USRS00.nDetections (blue curve) for the USRS00 sensor and Sensor.USonicRSI.Tx.USRS01.Rx.USRS01.nDetections (red curve) for the USRS01 sensor. These two parameters define the variation in obstacles identified by two ultrasonic sensors placed in different locations on the virtual vehicle model (Figure 43b). The variation in the vertical curves represents how many times each ultrasonic sensor identified an obstacle, object, or reflection during the time period studied.

The variance of the blue curve corresponds to the signals of the sensor framed in the blue square, whilst the variation in the red curve indicates the oscillations of the sensor’s signals framed in the red square.

If an ultrasonic sensor registers an increase in detections, it indicates that an obstacle has been recognized in the neighborhood; if the ultrasonic sensor detects nothing, it indicates that there may be blind zones around the virtual vehicle model.

## 5. Discussion

Regarding the future evolution of autonomous vehicles, Karp, L., noted in a report to the Stevens Institute of Technology [134] that assigning manufacturer liability for sensors and software algorithms will be a major driver of growth and innovation, given their critical role in ensuring the functional safety of such vehicles.

Replicating the behavior of real-world sensors in a virtual environment is a complex task. This involves capturing elements such as sensor noise, limitations in the field of view, uncertainty in physical parameters, and responses to varying environmental conditions. This can be achieved through advanced data-driven algorithms, AI-based simulation techniques, and innovative virtual sensor designs. Autonomous vehicles utilize a variety of sensors (cameras, radar, lidar, etc.), and combining data from multiple virtual and real sensors through sensor fusion techniques, each with its characteristics and potential errors, is a significant challenge [135]. Their performance can degrade due to changes in nonlinear dynamics, complex physical processes in the environment, and nonlinear interactions between input and output variables [136,137]. High-fidelity sensors can be computationally intensive to simulate real-world sensor data with high precision. This requires robust hardware and efficient algorithms to process the data in real-time [137]. Virtual sensors must operate in real time within the simulation environment, meaning they must generate and process data quickly enough to keep up with the simulation. This can be computationally demanding [138]. AI techniques enable the development of virtual sensors that can estimate physical parameters without traditional sensors, using data-driven models to emulate real-world conditions [11]. The integration of AI introduces potential uncertainties, necessitating validation to maintain trust in these systems [139].

Validating virtual sensor data against real-world data or physical sensor measurements can be difficult. Autonomous vehicles must handle unusual situations, and realistically simulating these circumstances adds another layer of complexity. Virtual sensors in the overall software architecture of autonomous vehicles can be complicated, mainly if the architecture was not designed initially with virtual sensors in mind. The development and validation of virtual sensors can be time-consuming and costly, especially when dealing with complex sensor models or high accuracy requirements.

Future studies could focus on improving the accuracy and robustness of virtual sensor models, particularly in complex and dynamic environments. This can address challenges related to nonlinear dynamics models with complex physical processes, research on environmental factors (such as weather conditions, temperature, and lighting), and detailed real-world behavioral models. Research into advanced AI and ML techniques for sensor fusion and data processing is also essential. Implementing these advancements is likely to result in more accurate and reliable data, thereby enhancing the overall performance of AV driving systems. Additionally, using DL architectures designed explicitly for particular tasks (like object detection or lane keeping) would be highly beneficial. Efforts should also concentrate on developing robust data-driven system models capable of accurately identifying various physical characteristics. Such models can provide a viable and cost-effective alternative to traditional real sensors. Research should include testing virtual sensor performance in rare or unexpected situations, often referred to as edge cases. This requires testing and validating across a wide range of scenarios.

The importance of developing virtual models of autonomous vehicles equipped with intelligent sensors is underscored by several international regulatory and legal frameworks governing automated driving systems:

UN Regulation No. 157 (2021/389) [140] introduces a multi-pillar certification framework for automated driving systems, including physical testing, audit procedures, and validated virtual simulations.

Regulation (EU) 2018/1592 [141] allows simulations to be used for vehicle feature evaluation, provided they accurately replicate real-world behavior.

ISO 26262—Functional Safety of Electrical/Electronic Systems [142] recommends simulation during development to identify and mitigate risks in electrical and electronic systems, ensuring compliance with safety standards.

ISO 34502:2022—Scenario-based Safety Evaluation Framework for Automated Driving Systems [143] presents a structured methodology for assessing safety performance in automated driving systems through standardized scenario libraries and virtual simulations.

ISO/PAS 21448:2022 Road Vehicles—Safety of the Intended Functionality [144] outlines guidelines for mitigating risks from functional limitations in automated systems, particularly in the absence of system failures, and emphasizes the role of scenario-based testing and simulation in evaluating real-world performance.

Validating and calibrating virtual sensors is also important and may involve establishing standardized testing procedures and metrics. Establishing industry standards for development and validation would promote interoperability and facilitate the widespread adoption of this technology. It is also crucial to optimize algorithms and ensure that hardware can meet the computational demands of high-fidelity sensor interfaces, especially in complex scenarios. Exploring hybrid approaches that combine both virtual sensor data and real sensor data could leverage the strengths of each type, thus enhancing overall accuracy and reliability. Also, it is important to examine the necessary levels of simulation fidelity for various autonomous driving tasks to balance the need for accuracy with computational efficiency. Studying how virtual sensor data are presented to human operators in semi-autonomous systems can also be an important step, as it impacts their situational awareness and decision-making processes.

## 6. Conclusions

This paper has explored the critical role of virtual sensors in the simulation and development of autonomous vehicles. Our study reveals that virtual sensors are not only substitutes for physical sensors but are integral components of advanced driver-assistance systems and autonomous driving functionalities. The classification of virtual sensors into three categories (ideal, Hi-Fi, and RSI) provides a structured approach to understanding their capabilities and limitations, demonstrating a range of fidelity from purely software-based abstractions to signal interfaces mimicking real-world sensor outputs.

Our study demonstrated that virtual sensors can effectively model various sensor functionalities, including slip angle, inertial measurements, object detection, and environmental perception. The examples provided show how these virtual sensors generate data that align with expected real-world behaviors, thus validating their role in simulation environments. The increasing complexity of sensor types, from ideal to RSI, highlights the trade-off between computational efficiency and simulation fidelity, a crucial consideration for real-time applications.

The implications of our research are significant. Accurately simulating a wide range of sensor data enables the development and testing of autonomous driving algorithms in a safe, controlled, and cost-effective virtual environment. This accelerates the development cycle by minimizing the need for physical testing and allowing the exploration of edge cases and rare scenarios that are difficult to replicate in the real world. Additionally, integrating AI and ML techniques into virtual sensor models opens new methods for adaptive and predictive sensing capabilities, enhancing the robustness and reliability of autonomous systems.

The advantages of virtual sensors include the following:

- Cost-effectiveness—virtual sensors are affordable alternatives to real sensors.
- Reduced vehicle mass—replacing physical sensors and associated wiring and connectors helps to reduce vehicle weight, leading to fuel and energy savings and lower emissions.
- Software-based—virtual sensors are developed using software applications and do not require additional hardware.
- Remote updates—firmware upgrades can be performed remotely via the Over-The-Air method, eliminating the need for physical interventions.
- Improved data accuracy and resolution—virtual sensors can improve data accuracy and resolution by merging information from multiple sources and by using data fusion algorithms.
- Expanded coverage—they can provide coverage to locations where physical sensors are unavailable.
- Preprocessing and optimization—when integrated into an embedded system, they can preprocess data, and perform error correction, merging, and optimization of input data.
- Flexibility—they can be simple or sophisticated, depending on the simulated activities and their consequences.
- Data accessibility—they can increase data accessibility from physical sensors, facilitating collaboration and efficient use of resources in interconnected systems like IoT.
- Reduced testing costs and time—using virtual sensors, developers can significantly reduce the cost and time associated with physical testing.
- Exploration of a wider range of scenarios—virtual sensors allow for exploring a wider range of scenarios and edge cases.

However, challenges remain. Replicating the nuanced behavior of real-world sensors, including noise, environmental dependencies, and physical limitations, requires sophisticated modeling techniques.

In conclusion, virtual sensors are indispensable tools in the development of autonomous vehicles. They facilitate comprehensive testing, accelerate development, and enable the exploration of advanced sensing capabilities.

## Figures and Tables

**Table 1:** The evolution of sensors depending on driving automation level.

Level 1		Level 2		Level 3		Level 4		Level 5 (Estimate)	
Model	Units	Model	Units	Model	Units	Model	Units	Model	Units
Ultrasonic	4	Ultrasonic	8	Ultrasonic	8	Ultrasonic	8	Ultrasonic	10
Radar long range	1	Radar long range	1	Radar long range	2	Radar long range	2	Radar long range	2
Radar short range	2	Radar short range	4	Radar short range	4	Radar short range	4	Radar short range	4
Camera mono	1	Camera mono	4	Camera mono	2	Camera mono	3	Camera mono	3
-	-	-	-	Camera stereo	1	Camera stereo	1	Camera stereo	2
-	-	-	-	Infra-red	1	Infra-red	1	Infra-red	2
-	-	-	-	Lidar 2D/3D	1	Lidar 2D/3D	4	Lidar 2D/3D	4
-	-	-	-	Global navigation	1	Global navigation	1	Global navigation	1
Total	8	Total	17	Total	20	Total	24	Total	28
2012		2016		2018		2020		Estimated by 2030	

**Table 2:** Road sensor functions.

Function	LK	LDW	AD	SD	EM	FC	WLD	PT
Road curvature								
Longitudinal/lateral slope								
Deviation angle/distance								
Lane information								
Road point position								
Road marker attributes								

The background color (gray) indicates which function corresponds (is active) to one of the listed systems (LK (Lane Keeping), LDW, AD (Autonomous Driving), SD (Sign Detection), EM (Energy Management), FC (Fuel Consumption), WLD (Wheel Lifting Detection), and PT (Powertrain)).

## Data Availability

Access to the data is available upon request. Access to the data can be requested via e-mail to the corresponding author.

## Abbreviations

The following abbreviations are used in this manuscript:

ABS	Antilock Braking System
ADAS	Advanced Driver Assistance System
ACC	Adaptive Cruise Control
AD	Autonomous driving
AEB	Automatic Emergency Braking
AI	Artificial Intelligence
AWD	All-Wheel Drive
BiSeNet	Bilateral Segmentation Network
CNN	Convolutional neural network
D-GNSS	Differential-Global Navigation Satellite System
CPU	Central Processing Unit
DL	Deep Learning
DMS	Driver Monitoring System
DPM	Deformable Part Model
DTw	Digital Twin
DVM	Double Validation Metric
EBA	Emergency Brake Assist
ECEF	Earth-Centered, Earth-Fixed
EDF	Empirical Cumulative Distribution Function
EM	Energy Management
ESC	Electronic Stability Control
FC	Fuel Consumption
FCW	Forward Collision Warning
FET	Field-Effect Transistor
FoV	Field-Of-View
GCS	Geographic Coordinate System
GDOP	Geometric Dilution of Precision
GNN	Global Nearest Neighbor
GNSS	Global Navigation Satellite System
GPS	Global Positioning System
GPU	Graphics Processing Unit
HD	High-Definition
Hi-Fi	High fidelity
HiL	Hardware-in-the-Loop
HV	High voltage
ID	Identifier
ILA	Intelligent Light Assist
IMU	Inertial Measurement Unit
IoT	Internet of Things
LDW	Lane Departure Warning
Lidar	Light detection and ranging
LK	Lane Keeping
LKA	Lane Keeping Assist
LV	Low voltage
MEMS	Micro-Electro-Mechanical Systems
ML	Machine Learning
MMS	Mobile Mapping System
OTA	Over-The-Air
PA	Park Assist
PPHT	Progressive Probabilistic Hough Transform
POI	Point Of Interest
PS	Physical sensors
PT	Powertrain
QTC	Quantum Tunneling Composite
RCS	Radar Cross-Section
RSI	Raw Signal Interface
RTK	Real-Time Kinematic
SAS	Smart Airbag System
SD	Sign Detection
SiL	Software-in-the-Loop
SLAM	Simultaneous localization And mapping
SNR	Signal-to-noise ratio
SPA	Sound Pressure Amplitude
SRTM	Shuttle Radar Topography Mission
ToF	Time of Flight
TSA	Traffic Sign Assist
V2I	Vehicle-to-Infrastructure
V2V	Vehicle-to-Vehicle
VDM	Vehicle dynamics model
VLC	Visible Light Communication
VS	Virtual sensors
WLD	Wheel Lifting Detection
YOLO	You Only Look Once

## References

[1] Martin D., Kühl N., Satzger G. (2021). Virtual Sensors. Bus. Inf. Syst. Eng..

[2] Dahiya R., Ozioko O., Cheng G. (2022). Sensory Systems for Robotic Applications.

[3] Šabanovič E., Kojis P., Šukevičius Š., Shyrokau B., Ivanov V., Dhaens M., Skrickij V. (2021). Feasibility of a Neural Network-Based Virtual Sensor for Vehicle Unsprung Mass Relative Velocity Estimation. Sensors.

[4] Persson J.A., Bugeja J., Davidsson P., Holmberg J., Kebande V.R., Mihailescu R.-C., Sarkheyli-Hägele A., Tegen A. (2023). The Concept of Interactive Dynamic Intelligent Virtual Sensors (IDIVS): Bridging the Gap between Sensors, Services, and Users through Machine Learning. Appl. Sci..

[5] Ambarish P., Mitradip B., Ravinder D. (2023). Solid-State Sensors.

[6] Shin H., Kwak Y. (2024). Enhancing digital twin efficiency in indoor environments: Virtual sensor-driven optimization of physical sensor combinations. Automat. Constr..

[7] Stanley M., Lee J. (2018). Sensor Analysis for the Internet of Things.

[8] Stetter R. (2020). A Fuzzy Virtual Actuator for Automated Guided Vehicles. Sensors.

[9] Xie J., Yang R., Gooi H.B., Nguyen H. (2023). PID-based CNN-LSTM for accuracy-boosted virtual sensor in battery thermal management system. Appl. Energ..

[10] Iclodean C., Cordos N., Varga B.O. (2020). Autonomous Shuttle Bus for Public Transportation: A Review. Energies.

[11] Fabiocchi D., Giulietti N., Carnevale M., Giberti H. (2024). AI-Driven Virtual Sensors for Real-Time Dynamic Analysis of Me-chanisms: A Feasibility Study. Machines.

[12] Kabadayi S., Pridgen A., Julien C. Virtual sensors: Abstracting data from physical sensors. Proceedings of the IEEE International Symposium on a World of Wireless, Mobile and Multimedia Networks.

[13] Compredict. https://compredict.ai/virtual-sensors-replacing-vehicle-hardware-sensors/.

[14] Prokhorov D. Virtual Sensors and Their Automotive Applications. Proceedings of the 2005 International Conference on Intelligent Sensors, Sensor Networks and Information Processing.

[15] Forssell U., Ahlqvist S., Persson N., Gustafsson F., Krueger S., Gessner W. (2012). Virtual Sensors for Vehicle Dynamics Applications. Advanced Microsystems for Automotive Applications 2001.

[16] Hu X.H., Cao L., Luo Y., Chen A., Zhang E., Zhang W. (2019). A Novel Methodology for Comprehensive Modeling of the Kinetic Behavior of Steerable Catheters. IEEE/ASME Trans. Mechatron..

[17] Cummins. https://www.cummins.com/news/2024/03/18/virtual-sensors-and-their-role-energy-future.

[18] Bucaioni A., Pelliccione P., Mubeen S. (2024). Modelling centralised automotive E/E software architectures. Adv. Eng. Inform..

[19] Zhang Q., Shen S., Li H., Cao W., Tang W., Jiang J., Deng M., Zhang Y., Gu B., Wu K. (2022). Digital twin-driven intelligent production line for automotive MEMS pressure sensors. Adv. Eng. Inform..

[20] Ida N. (2020). Sensors, Actuators, and Their Interfaces: A multidisciplinary Introduction.

[21] Masti D., Bernardini D., Bemporad A. (2021). A machine-learning approach to synthesize virtual sensors for parameter-varying systems. Eur. J. Control..

[22] Paepae T., Bokoro P.N., Kyamakya K. (2021). From fully physical to virtual sensing for water quality assessment: A comprehensive review of the relevant state-of-the-art. Sensors.

[23] Tihanyi V., Tettamanti T., Csonthó M., Eichberger A., Ficzere D., Gangel K., Hörmann L.B., Klaffenböck M.A., Knauder C., Luley P. (2021). Motorway Measurement Campaign to Support R&D Activities in the Field of Automated Driving Technologies. Sensors.

[24] Tactile Mobility. https://tactilemobility.com/.

[25] Compredict-Virtual Sensor Platform. https://compredict.ai/virtual-sensor-platform/.

[26] Mordor Intellingence. https://www.mordorintelligence.com/industry-reports/virtual-sensors-market.

[27] Iclodean C., Varga B.O., Cordoș N. (2022). Autonomous Driving Technical Characteristics. Autonomous Vehicles for Public Transportation, Green Energy and Technology.

[28] SAE. https://www.sae.org/standards/content/j3016_202104/.

[29] Muhovič J., Perš J. (2020). Correcting Decalibration of Stereo Cameras in Self-Driving Vehicles. Sensors.

[30] Hamidaoui M., Talhaoui M.Z., Li M., Midoun M.A., Haouassi S., Mekkaoui D.E., Smaili A., Cherraf A., Benyoub F.Z. (2025). Survey of Autonomous Vehicles’ Collision Avoidance Algorithms. Sensors.

[31] Cabon Y., Murray N., Humenberger M. (2020). Virtual KITTI 2. arXiv.

[32] Mallik A., Gaopande M.L., Singh G., Ravindran A., Iqbal Z., Chao S., Revalla H., Nagasamy V. (2022). Real-time Detection and Avoidance of Obstacles in the Path of Autonomous Vehicles Using Monocular RGB Camera. SAE Int. J. Adv. Curr. Pract. Mobil..

[33] Zhe T., Huang L., Wu Q., Zhang J., Pei C., Li L. (2020). Inter-Vehicle Distance Estimation Method Based on Monocular Vision Using 3D Detection. IEEE Trans. Veh. Technol..

[34] Rill R.A., Faragó K.B. (2021). Collision avoidance using deep learning-based monocular vision. SN Comput. Sci..

[35] He J., Tang K., He J., Shi J. (2020). Effective vehicle-to-vehicle positioning method using monocular camera based on VLC. Opt. Express.

[36] Choi W.Y., Yang J.H., Chung C.C. (2021). Data-Driven Object Vehicle Estimation by Radar Accuracy Modeling with Weighted Interpolation. Sensors.

[37] Muckenhuber S., Museljic E., Stettinger G. (2022). Performance evaluation of a state-of-the-art automotive radar and corres-ponding modeling approaches based on a large labeled dataset. J. Intell. Transport. Syst..

[38] Sohail M., Khan A.U., Sandhu M., Shoukat I.A., Jafri M., Shin H. (2023). Radar sensor based Machine Learning approach for precise vehicle position estimation. Sci. Rep..

[39] Srivastav A., Mandal S. (2023). Radars for autonomous driving: A review of deep learning methods and challenges. IEEE Access.

[40] Poulose A., Baek M., Han D.S. Point cloud map generation and localization for autonomous vehicles using 3D lidar scans. Proceedings of the 2022 27th Asia Pacific Conference on Communications (APCC).

[41] Saha A., Dhara B.C. (2024). 3D LiDAR-based obstacle detection and tracking for autonomous navigation in dynamic environments. Int. J. Intell. Robot. Appl..

[42] Dazlee N.M.A.A., Khalil S.A., Rahman S.A., Mutalib S. (2022). Object detection for autonomous vehicles with sensor-based technology using YOLO. Int. J. Intell. Syst. Appl. Eng..

[43] Guan L., Chen Y., Wang G., Lei X. (2020). Real-time vehicle detection framework based on the fusion of LiDAR and camera. Electronics.

[44] Kotur M., Lukić N., Krunić M., Lukač Ž. Camera and LiDAR sensor fusion for 3d object tracking in a collision avoidance system. Proceedings of the 2021 Zooming Innovation in Consumer Technologies Conference (ZINC).

[45] Choi W.Y., Kang C.M., Lee S.H., Chung C.C. (2020). Radar accuracy modeling and its application to object vehicle tracking. Int. J. Control. Autom. Syst..

[46] Simcenter. https://blogs.sw.siemens.com/simcenter/the-sense-of-virtual-sensors/.

[47] Kim J., Kim Y., Kum D. Low-level sensor fusion network for 3D vehicle detection using radar range-azimuth heatmap and monocular image. Proceedings of the Asian Conference on Computer Vision.

[48] Lim S., Jung J., Lee B.H., Choi J., Kim S.C. (2022). Radar sensor-based estimation of vehicle orientation for autonomous driving. IEEE Sensors J..

[49] Caesar H., Bankiti V., Lang A.H., Vora S., Liong V.E., Xu Q., Krishnan A., Pan Y., Baldan G., Beijbom O. nuScenes: A multimodal dataset for autonomous driving. Proceedings of the IEEE/CVF Conference on Computer Vision and Pattern Recognition.

[50] Robsrud D.N., Øvsthus Ø., Muggerud L., Amendola J., Cenkeramaddi L.R., Tyapin I., Jha A. Lidar-mmW Radar Fusion for Safer UGV Autonomous Navigation with Collision Avoidance. Proceedings of the 2023 11th International Conference on Control, Mechatronics and Automation (ICCMA).

[51] Wang Y., Jiang Z., Gao X., Hwang J.N., Xing G., Liu H. RODnet: Radar object detection using cross-modal supervision. Proceedings of the IEEE/CVF Winter Conference on Applications of Computer Vision.

[52] Rövid A., Remeli V., Paufler N., Lengyel H., Zöldy M., Szalay Z. (2020). Towards Reliable Multisensory Perception and Its Automotive Applications. Period. Polytech. Transp. Eng..

[53] IPG, CarMaker. https://www.ipg-automotive.com/en/products-solutions/software/carmaker/.

[54] Yeong D.J., Velasco-Hernandez G., Barry J., Walsh J. (2021). Sensor and Sensor Fusion Technology in Autonomous Vehicles: A Review. Sensors.

[55] Liu X., Baiocchi O. A comparison of the definitions for smart sensors, smart objects and Things in IoT. Proceedings of the 2016 IEEE 7th Annual Information Technology, Electronics and Mobile Communication Conference (IEMCON).

[56] Peinado-Asensi I., Montés N., García E. (2023). Virtual Sensor of Gravity Centres for Real-Time Condition Monitoring of an Industrial Stamping Press in the Automotive Industry. Sensors.

[57] Stetter R., Witczak M., Pazera M. (2018). Virtual Diagnostic Sensors Design for an Automated Guided Vehicle. Appl. Sci..

[58] Lengyel H., Maral S., Kerebekov S., Szalay Z., Török Á. (2023). Modelling and simulating automated vehicular functions in critical situations—Application of a novel accident reconstruction concept. Vehicles.

[59] Dörr D. (2017). Using Virtualization to Accelerate the Development of ADAS & Automated Driving Functions.

[60] Kim J., Park S., Kim J., Yoo J. (2023). A Deep Reinforcement Learning Strategy for Surrounding Vehicles-Based Lane-Keeping Control. Sensors.

[61] Pannagger P., Nilac D., Orucevic F., Eichberger A., Rogic B. (2021). Advanced Lane Detection Model for the Virtual Development of Highly Automated Functions. arXiv.

[62] IPG Automotive (2023). IPG Guide-User’s Guide Version 12.0.1 CarMaker.

[63] Iclodean C., Varga B.O., Cordoș N. (2022). Virtual Model. Autonomous Vehicles for Public Transportation, Green Energy and Technology.

[64] Schäferle S. (2019). Choosing the Correct Sensor Model for Your Application. IPG Automotive. https://www.ipg-automotive.com/uploads/tx_pbfaqtickets/files/98/SensorModelLevels.pdf.

[65] Magosi Z.F., Wellershaus C., Tihanyi V.R., Luley P., Eichberger A. (2022). Evaluation Methodology for Physical Radar Perception Sensor Models Based on On-Road Measurements for the Testing and Validation of Automated Driving. Energies.

[66] IPG Automotive (2023). Reference Manual Version 12.0.1 CarMaker.

[67] Iclodean C. (2023). Introducere în Sistemele Autovehiculelor.

[68] Renard D., Saddem R., Annebicque D., Riera B. (2024). From Sensors to Digital Twins toward an Iterative Approach for Existing Manufacturing Systems. Sensors.

[69] Brucherseifer E., Winter H., Mentges A., Mühlhäuser M., Hellmann M. (2021). Digital Twin conceptual framework for improving critical infrastructure resilience. at-Automatisierungstechnik.

[70] Grieves M., Vickers J. (2016). Digital twin: Mitigating unpredictable, undesirable emergent behavior in complex systems. Transdisciplinary Perspectives on Complex Systems: New Findings and Approaches.

[71] Kritzinger W., Karner M., Traar G., Henjes J., Sihn W. (2018). Digital Twin in manufacturing: A categorical literature review and classification. Ifac-PapersOnline.

[72] Shoukat M.U., Yan L., Yan Y., Zhang F., Zhai Y., Han P., Nawaz S.A., Raza M.A., Akbar M.W., Hussain A. (2024). Autonomous driving test system under hybrid reality: The role of digital twin technology. Internet Things.

[73] Tu L., Xu M. (2024). An Analysis of the Use of Autonomous Vehicles in the Shared Mobility Market: Opportunities and Challenges. Sustainability.

[74] Navya Brochure-Autonom-Shuttle-Evo. https://navya.tech/wp-content/uploads/documents/Brochure-Autonom-Shuttle-Evo-EN.pdf.

[75] Navya Self-Driving Shuttle for Passenger Transportation. https://www.navya.tech/en/solutions/moving-people/self-driving-shuttle-for-passenger-transportation/.

[76] Patentimage. https://patentimages.storage.googleapis.com/12/0f/d1/33f8d2096f49f6/US20180095473A1.pdf.

[77] AVENUE Autonomous Vehicles to Evolve to a New Urban Experience Report. https://h2020-avenue.eu/wp-content/uploads/2023/03/Keolis-LyonH2020-AVENUE_Deliverable_7.6_V2-not-approved.pdf.

[78] EarthData Search. https://search.earthdata.nasa.gov/search?q=SRTM.

[79] GpsPrune. https://activityworkshop.net/software/gpsprune/download.html.

[80] IPG Automotive (2023). InfoFile Description Version 12.0.1 IPGRoad.

[81] IPG Automotive (2023). User Manual Version 12.0.1 IPGDriver.

[82] Piyabongkarn D.N., Rajamani R., Grogg J.A., Lew J.Y. (2009). Development and Experimental Evaluation of a Slip Angle Estimator for Vehicle Stability Control. IEEE Trans. Control. Syst. Technol..

[83] IPG Automotive (2023). CarMaker Reference Manual 12.0.2 CarMaker.

[84] Pacejka H.B. (2006). Tyre and Vehicle Dynamics.

[85] Salminen H. (2014). Parametrizing Tyre Wear Using a Brush Tyre Model. Master’s Thesis.

[86] Pacjka H.B., Besselink I.J.M. (1997). Magic Formula Tyre Model with Transient Properties. Veh. Syst. Dyn..

[87] Pacejka H.B., Pacejka H.B. (2012). Chapter 4—Semi-Empirical Tire Models. Tire and Vehicle Dynamics.

[88] Guo Q., Xu Z., Wu Q., Duan J. The Application of in-the-Loop Design Method for Controller. Proceedings of the 2nd IEEE Conference on Industrial Electronics and Applications.

[89] Chen T., Chen L., Xu X., Cai Y., Jiang H., Sun X. (2019). Sideslip Angle Fusion Estimation Method of an Autonomous Electric Vehicle Based on Robust Cubature Kalman Filter with Redundant Measurement Information. World Electr. Veh. J..

[90] Jin L., Xie X., Shen C., Wang F., Wang F., Ji S., Guan X., Xu J. (2017). Study on electronic stability program control strategy based on the fuzzy logical and genetic optimization method. Adv. Mech. Eng..

[91] Zhao Z., Chen H., Yang J., Wu X., Yu Z. (2014). Estimation of the vehicle speed in the driving mode for a hybrid electric car based on an unscented Kalman filter. Proc. Inst. Mech. Eng. Part D J. Automob. Eng..

[92] Li Q., Chen L., Li M., Shaw S.-L., Nuchter A. (2013). A Sensor-Fusion Drivable-Region and Lane-Detection System for Auto-nomous Vehicle Navigation in Challenging Road Scenarios. IEEE Trans. Veh. Technol..

[93] Rana M.M. (2017). Attack Resilient Wireless Sensor Networks for Smart Electric Vehicles. IEEE Sens. Lett..

[94] Xia X., Xiong L., Huang Y., Lu Y., Gao L., Xu N., Yu Z. (2022). Estimation on IMU yaw misalignment by fusing information of automotive onboard sensors. Mech. Syst. Signal Process..

[95] Sieberg P.M., Schramm D. (2022). Ensuring the Reliability of Virtual Sensors Based on Artificial Intelligence within Vehicle Dynamics Control Systems. Sensors.

[96] Xiong L., Xia X., Lu Y., Liu W., Gao L., Song S., Han Y., Yu Z. (2019). IMU-Based Automated Vehicle Slip Angle and Attitude Estimation Aided by Vehicle Dynamics. Sensors.

[97] Ess A., Schindler K., Leibe B., Van Gool L. (2010). Object detection and tracking for autonomous navigation in dynamic environments. Int. J. Robot. Res..

[98] Bewley A., Ge Z., Ott L., Ramos F., Upcroft B. Simple online and realtime tracking. Proceedings of the 2016 IEEE International Conference on Image Processing (ICIP).

[99] Banerjee S., Serra J.G., Chopp H.H., Cossairt O., Katsaggelos A.K. An Adaptive Video Acquisition Scheme for Object Tracking. Proceedings of the 27th European Signal Processing Conference (EUSIPCO).

[100] Chen N., Li M., Yuan H., Su X., Li Y. (2021). Survey of pedestrian detection with occlusion. Complex Intell. Syst..

[101] Liu Z., Chen W., Wu X. Salient region detection using high level feature. Proceedings of the 13th International Conference on Control Automation Robotics & Vision (ICARCV).

[102] Felzenszwalb P., Girshick R., McAllester D., Ramanan D. (2013). Visual object detection with deformable part models. Commun. ACM.

[103] Kato S., Takeuchi E., Ishiguro Y., Ninomiya Y., Takeda K., Hamada T. (2015). An Open Approach to Autonomous Vehicles. IEEE Micro.

[104] Broggi A., Cattani S., Patander M., Sabbatelli M., Zani P. A full-3D voxel-based dynamic obstacle detection for urban scenario using stereo vision. Proceedings of the 16th International IEEE Conference on Intelligent Transportation Systems (ITSC 2013).

[105] Patra S., Maheshwari P., Yadav S., Arora C., Banerjee S. (2018). A Joint 3D-2D based Method for Free Space Detection on Roads. arXiv.

[106] Vitor G.B., Lima D.A., Victorino A.C., Ferreira J.V. A 2D/3D vision based approach applied to road detection in urban environments. Proceedings of the IEEE Intelligent Vehicles Symposium (IV 2013).

[107] Heinz L. (2019). CarMaker Tips & Tricks No. 3-011 Detect Traffic Lights.

[108] Zhang P., Zhang M., Liu J. (2021). Real-time HD map change detection for crowdsourcing update based on mid-to-high-end sensors. Sensors.

[109] Bahlmann C., Zhu Y., Ramesh V., Pellkofer M., Koehler T. A System for Traffic Sign Detection, Tracking, and Recognition Using Color, Shape, and Motion Information. Proceedings of the IEEE Proceedings of Intelligent Vehicles Symposium.

[110] Fazekas Z., Gerencsér L., Gáspár P. (2021). Detecting Change between Urban Road Environments along a Route Based on Static Road Object Occurrences. Appl. Sci..

[111] Liu C., Tao Y., Liang J., Li K., Chen Y. Object detection based on YOLO network. Proceedings of the 2018 IEEE 4th In-formation Technology and Mechatronics Engineering Conference (ITOEC).

[112] Nuthong C., Charoenpong T. Lane Detection using Smoothing Spline. Proceedings of the 3rd International Congress on Image and Signal Processing.

[113] Dou J., Li J. (2013). Robust object detection based on deformable part model and improved scale invariant feature transform. Optik.

[114] Lindenmaier L., Aradi S., Bécsi T., Törő O., Gáspár P. (2023). Object-Level Data-Driven Sensor Simulation for Automotive Environment Perception. IEEE Trans. Intell. Veh..

[115] Bird J., Bird J. (2006). Higher Engineering Mathematics.

[116] Ainsalu J., Arffman V., Bellone M., Ellner M., Haapamäki T., Haavisto N., Josefson E., Ismailogullari A., Lee B., Ma-dland O. (2018). State of the Art of Automated Buses. Sustainability.

[117] Lian H., Li M., Li T., Zhang Y., Shi Y., Fan Y., Yang W., Jiang H., Zhou P., Wu H. (2025). Vehicle speed measurement method using monocular cameras. Sci. Rep..

[118] Vivacqua R., Vassallo R., Martins F. (2017). A Low Cost Sensors Approach for Accurate Vehicle Localization and Autonomous Driving Application. Sensors.

[119] Xue L., Li M., Fan L., Sun A., Gao T. (2021). Monocular Vision Ranging and Camera Focal Length Calibration. Sci. Program..

[120] Kerbl B., Kopanas G., Leimkuhler T., Drettakis G. (2023). 3D Gaussian Splatting for Real-Time Radiance Field Rendering. ACM Trans. Graph..

[121] Santosh Reddy P., Abhiram H., Archish K.S. A Survey of 3D Gaussian Splatting: Optimization Techniques, Applications, and AI-Driven Advancements. Proceedings of the 2025 International Conference on Intelligent and Innovative Technologies in Computing, Electrical and Electronics (IITCEE).

[122] Qiu S., Xie B., Liu Q., Heng P.-A. Creating Virtual Environments with 3D Gaussian Splatting: A Comparative Study. Proceedings of the 2025 IEEE Conference on Virtual Reality and 3D User Interfaces Abstracts and Workshops (VRW).

[123] Hornáček M., Rozinaj G. Exploring 3D Gaussian Splatting: An Algorithmic Perspective. Proceedings of the 2024 International Symposium ELMAR.

[124] Rosique F., Navarro P.J., Fernández C., Padilla A. (2019). A Systematic Review of Perception System and Si-mulators for Autonomous Vehicles Research. Sensors.

[125] Elster L., Staab J.P., Peters S. (2023). Making Automotive Radar Sensor Validation Measurements Comparable. Appl. Sci..

[126] Roy C.J., Balch M.S. (2021). A Holistic Approach to Uncertainty Quantification with Application to Supersonic Nozzle Thrust. Int. J. Uncertain. Quantif..

[127] Magosi Z.F., Eichberger A. (2023). A Novel Approach for Simulation of Automotive Radar Sensors Designed for Systematic Support of Vehicle Development. Sensors.

[128] Maier M., Makkapati V.P., Horn M. Adapting Phong into a Simulation for Stimulation of Automotive Radar Sensors. Proceedings of the 2018 IEEE MTT-S International Conference on Microwaves for Intelligent Mobility (ICMIM).

[129] Minin I.V., Minin O.V. (2008). Lens Candidates to Antenna Array. Basic Principles of Fresnel Antenna Arrays.

[130] Sensors Partners LiDAR Laser: What Is LiDAR and How Does It Work?|Sensor Partners. https://sensorpartners.com/en/knowledge-base/how-a-lidar-laser-works/.

[131] García-Gómez P., Royo S., Rodrigo N., Casas J.R. (2020). Geometric Model and Calibration Method for a Solid-State LiDAR. Sensors.

[132] Kim G. (2022). Performance Index for Extrinsic Calibration of LiDAR and Motion Sensor for Mapping and Localization. Sensors.

[133] Schmoll L., Kemper H., Hagenmüller S., Brown C.L. (2024). Validation of an Ultrasonic Sensor Model for Application in a Simulation Platform. ATZelectronics Worldw..

[134] Stevens Institude of Thechnology 1870. https://www.stevens.edu/news/autonomous-vehicles-will-add-us81-billion-new-premiums-auto-insurers-2025-according-accenture-report.

[135] Sen S., Husom E.J., Goknil A., Tverdal S., Nguyen P. (2024). Uncertainty-Aware Virtual Sensors for Cyber-Physical Systems. IEEE Softw..

[136] Ying Z., Wang Y., He Y., Wang J. (2022). Virtual Sensing Techniques for Nonlinear Dynamic Processes Using Weighted Proba-bility Dynamic Dual-Latent Variable Model and Its Industrial Applications. Knowl.-Based Syst..

[137] Yuan X., Rao J., Wang Y., Ye L., Wang K. (2022). Virtual Sensor Modeling for Nonlinear Dynamic Processes Based on Local Weighted PSFA. IEEE Sens. J..

[138] Zheng T. (2023). Algorithmic Sensing: A Joint Sensing and Learning Perspective. Proceedings of the 21st Annual International Conference on Mobile Systems, Applications and Services.

[139] Es-haghi M.S., Anitescu C., Rabczuk T. (2024). Methods for Enabling Real-Time Analysis in Digital Twins: A Literature Review. Comput. Struct..

[140] EUR-Lex, UN Regulation No 157—Uniform Provisions Concerning the Approval of Vehicles with Regards to Automated Lane Keeping Systems [2021/389]. https://eur-lex.europa.eu/eli/reg/2021/389/oj/eng.

[141] EUR-Lex, Regulation No 140 of the Economic Commission for Europe of the United Nations (UN/ECE)—Uniform Provisions Concerning the Approval of Passenger Cars with Regard to Electronic Stability Control (ESC) Systems [2018/1592]. https://eur-lex.europa.eu/eli/reg/2018/1592/oj/eng.

[142] International Organization for Standardization ISO 26262-1:2018(en) Road Vehicles—Functional Safety—Part 1: Vocabulary. https://www.iso.org/obp/ui/en/#iso:std:iso:26262:-1:ed-2:v1:en.

[143] International Organization for Standardization ISO 34502:2022 Road Vehicles—Test Scenarios for Automated Driving Systems—Scenario Based Safety Evaluation Framework. https://www.iso.org/standard/78951.html.

[144] International Organization for Standardization ISO 21448:2022(en); Road Vehicles—Safety of the Intended Functionality. https://www.iso.org/obp/ui/en/#iso:std:iso:21448:ed-1:v1:en.

